# Network-Based Analysis Reveals Functional Connectivity Related to Internet Addiction Tendency

**DOI:** 10.3389/fnhum.2016.00006

**Published:** 2016-02-01

**Authors:** Tanya Wen, Shulan Hsieh

**Affiliations:** ^1^Cognitive Electrophysiology Lab: Control, Aging, Sleep, and Emotion, Department of Psychology, National Cheng Kung UniversityTainan, Taiwan; ^2^Department of Life Sciences, National Cheng Kung UniversityTainan, Taiwan; ^3^Institute of Allied Health Sciences, National Cheng Kung UniversityTainan, Taiwan; ^4^Department of Public Health, National Cheng Kung UniversityTainan, Taiwan

**Keywords:** internet addiction, network-based statistics, functional connectivity, resting-state, meta-analysis

## Abstract

Preoccupation and compulsive use of the internet can have negative psychological effects, such that it is increasingly being recognized as a mental disorder. The present study employed network-based statistics to explore how whole-brain functional connections at rest is related to the extent of individual’s level of internet addiction, indexed by a self-rated questionnaire. We identified two topologically significant networks, one with connections that are positively correlated with internet addiction tendency, and one with connections negatively correlated with internet addiction tendency. The two networks are interconnected mostly at frontal regions, which might reflect alterations in the frontal region for different aspects of cognitive control (i.e., for control of internet usage and gaming skills). Next, we categorized the brain into several large regional subgroupings, and found that the majority of proportions of connections in the two networks correspond to the cerebellar model of addiction which encompasses the four-circuit model. Lastly, we observed that the brain regions with the most inter-regional connections associated with internet addiction tendency replicate those often seen in addiction literature, and is corroborated by our meta-analysis of internet addiction studies. This research provides a better understanding of large-scale networks involved in internet addiction tendency and shows that pre-clinical levels of internet addiction are associated with similar regions and connections as clinical cases of addiction.

## Introduction

Internet addiction (OReilly, 1996; Young, 1998) is a modern phenomenon that is characterized by preoccupation and compulsive use of the internet. In particular, internet gaming disorder (IGD) has been listed in Section III of the Diagnostic and Statistical Manual version 5 (DSM-5^®^, American Psychiatric Association [APA], 2013). Due to a lack of a standard criterion, some literature treated the two terminology as synonymous (see Petry and O’Brien, 2013; Petry et al., 2014 for a discussion); however, the compulsive and excessive use of the internet for any activity (which we will refer to in this literature as internet addiction) is more global than its major subtype IGD, which can include multiple forms of internet use in addition to online gaming (Griffiths and Pontes, 2014; Király et al., 2014; Petry et al., 2014). Our current study investigates internet addiction in the more general form. Similar to substance use disorders, internet addiction shows withdrawal symptoms, tolerance, loss of control, and psychosocial problems, leading to clinically significant distress or impairment in daily functioning. Prevalence seems highest Asian countries and in male adolescents, and has been estimated to range from 14.1 to 16.5% (95 percent confidence interval) among Taiwan college students in one study (Lin et al., 2011). The phenomenon has been attracting more attention over the past few years and clearly deserves further research.

Functional magnetic resonance imaging (fMRI) has been employed to identify the neural substrates of internet addiction, which turned out to show similar brain signatures with substance-related addictions (Kuss and Griffiths, 2012; Brand et al., 2014; Meng et al., 2015). In blocked and event-related studies, several regions associated with reward, addiction, and craving have been identified by contrasting internet gaming cues with baseline, which includes the insula, nucleus accumbens (NAc), dorsolateral prefrontal cortex (DLPFC), and orbital frontal cortex (OFC) (Hoeft et al., 2008; Ko et al., 2009; Han et al., 2010; Sun et al., 2012; Ko et al., 2013). However, activation-based approaches contrast cue-related activity and do not address how regions of the brain interact, and thus cannot characterize altered functional connections associated with clinical or behavioral measures; yet human disorders are a result of disturbances in an interconnected complex system (Fornito and Bullmore, 2015). The introduction of resting-state fMRI has proved to be a powerful tool for studying whole brain neural connectivity (van den Heuvel and Pol, 2010). Resting-state functional connectivity is assessed by the correlation of spontaneous fluctuations of blood oxygen level-dependent (BOLD) signals in different regions of the brain and is thought to provide a measure of its functional organization, and can help characterize abnormal synchronizations between brain regions in the spectrum of psychological phenotypes (Biswal et al., 2010; Craddock et al., 2013).

Although there have been some studies that have employed functional connectivity to investigate altered functional connectivity associated with internet addiction, most studies used seed regions chosen a priori, either (a) correlating one seed region with the remaining voxels of the whole brain [Hoeft et al., 2008 used the NAc; Lorenz et al., 2013 used the right inferior frontal gyrus (IFG); Ding et al., 2013 used the posterior cingulate cortex (PCC); Ko et al., 2015 used the amygdala; Zhang et al., 2015 used the insula; Hong et al., 2015 used the caudate nucleus and putamen; Kühn and Gallinat, 2015 used the right frontal pole; Li et al., 2015 used the right DLPFC] or (b) performing correlations among multiple predefined ROIs selected from meaningful networks (Yuan et al., 2015 examined the central executive network and salience network; Dong et al., 2015b examined the executive control network; Dong et al., 2015a examined the executive control network and reward network; Li et al., 2014 examined the response inhibition network; Lin et al., 2015 examined six predefined bilateral corticostriatal ROIs). The pre-defined seed regions examined only represent a small proportion of the brain, thus they may not be able to provide a complete picture of how the connectome is affected by internet addiction.

Very few studies have used a whole-brain approach to study internet addiction. To our knowledge, there are currently only four published papers that adopted a whole-brain approach, and their methods are quite variable, ranging from network-based statistics (NBS; Hong et al., 2013) to topological (Hong et al., 2013; Wee et al., 2014; Park et al., 2015) to a novelly developed voxel-mirrored homotopic connectivity (Wang et al., 2015). In particular, Hong et al. (2013) employed NBS to identify between-group differences in inter-regional functional connectivity, and found impaired connections involved in cortico-subcortical circuits in patients with internet addiction. However, their study focused on a small sample size of a unique population (male early adolescents).

Therefore, in our current paper, we decided to use a whole-brain connectivity approach, NBS (Zalesky et al., 2010a; Han et al., 2013), to identify functional connections that are predictive of internet addiction tendency. NBS is a validated statistical method to deal with the multiple comparisons problem on a graph, it is analogous to cluster-based methods (Nichols and Holmes, 2002), and is used to identify connections and networks comprising the human connectome that are associated with an experimental effect or a between-group difference by testing the hypothesis independently at every connection. Our results will furthermore be compared with a meta-analysis of existing papers related to the neural correlates of internet addiction. We hope to extend the existing literature in several ways: (1) We hope to provide a more complete picture of internet addiction by using whole-brain analysis instead of using only a small number of pre-defined seed regions. (2) Although there exists a couple of whole-brain functional connectivity studies on internet addiction (e.g., Hong et al., 2013; Wang et al., 2015), the studies compared internet addiction groups with healthy controls. Our study did not involve any clinical patients, but characterized internet addiction tendency as a gradient. We hope to identify functional connections whose strength is modulated by level of addiction. (3) Most internet addiction studies have not taken the cerebellum into consideration, yet the cerebellum has been implicated as an important region in addiction (Moulton et al., 2014). Thus, we have included the cerebellum in our analysis. (4) Many studies have limited their participant group to males, and often contain relatively small sample sizes (e.g., Hong et al., 2013, 2015; Kühn and Gallinat, 2015). To increase the generalizability and power of these studies, samples containing both genders and a larger sample size is necessary (Li et al., 2015). By tackling the above problems, the current study hopes to provide a better understanding of how functional connectivity is associated with internet addiction tendency.

## Materials and Methods

### Meta-Analysis

A meta-analysis was constructed using the NeuroSynth database (http://neurosynth.org; Yarkoni et al., 2011). A customized analysis was performed by using the search terms “addiction,” “addict,” “internet,” “gaming,” “game,” and “online” to identify studies related to internet addiction in the database. The criteria of inclusion was verified manually, and a list of the studies included are detailed in the Supplementary Materials 1. A total of 18 studies were included. Peak activation coordinates as well as its neighborhood of 6 mm voxels were extracted from the included studies. Next, a meta-analysis of these coordinates was performed, producing forward and revere inference whole-brain *z*-score maps. The forward inference maps reflect the likelihood that a region will activate given these terms [*P*(activation| terms)], therefore informing us of the consistency of activation for the given terms. The reverse inference map shows the likelihood that these terms are used in a study given the presence of reported activation [*P*(terms| activation)]; thus a region that is activated indicates it is more likely to be an internet addiction related study than a non-internet addiction related study, reflecting selectivity of that region. Since both forward and reverse inference play an important role in helping us understand regions associated with internet addiction, we overlapped these two inference maps to outline their common regions. Clusters greater than five voxels are reported.

### Resting-State fMRI

#### Participants

Forty-seven healthy participants (21 males and 26 females) from southern Taiwan, most of which are students or staff in the university, were recruited through advertisements, to participate in the experiment (age range = 19–29 years, mean age = 22.87 years, *SD* = 2.22 years). The participants were right-handed (indicated by the Edinburgh Handedness Inventory), had normal or corrected-to-normal vision, and no history of psychological or neural disorders. Their depression, anxiety and intelligence scores were in the normal range [Beck’s Depression Inventory (BDI) score: 0–12; Beck’s Anxiety Inventory (BAI) score: 0–7; Raven’s Standard Progressive Matrices test score: 35–57]. The Chen Internet Addiction Scale-Revised (CIAS-R) score of all participants had range = 28–92, mean = 60.04, *SD* = 16.53. **Table 1** summarizes the demographic information and behavioral characteristics of the participants. The normality of the CIAS-R scores was verified by the Shapiro–Wilk test [*W*(47) = 0.98, *p* = 0.50]. There was no significant correlation between gender and CIAS-R score (Spearman’s ρ = 0.15, *p* = 0.30). All participants provided their written informed consent, and the study protocol was approved (NO: B-ER-101-144) by the Institutional Review Board (IRB) of the National Cheng Kung University Hospital, Tainan, Taiwan. All participants were paid 500 NTD after completion of the experiment.

**Table 1:** Demographic information and behavioral characteristics.

	Range	Mean	SD
Age	19–29	22.87	2.22
BDI	0–12	4.17	3.74
BAI	0–7	1.81	2.02
SPM	35–57	46.28	5.19
CIAS-R	28–92	60.04	16.53


*BDI, Beck’s Depression Inventory; BAI, Beck,s Anxiety Inventory; SPM, Raven’s Standard Progressive Matrices; CIAS-R, Chen Internet Addiction Scale-Revised.

#### Chen Internet Addiction Scale-Revised (CIAS-R) Questionnaire

The Chen Internet Addiction Scale-Revised (CIAS-R; Chen et al., 2003) is a 26-item measure used to assess the severity of internet addiction. The CIAS-R is based on the DSM-IV-TR additive behaviors criteria and contains two subscales of internet addiction (a) core symptoms and (b) related problems, assessing five dimensions including (1) compulsive internet use, (2) withdrawal symptoms when the internet is taken away, (3) tolerance, (4) jeopardy of interpersonal relationships and physical health, and (5) time management problems. Items are rated on a 4-point Likert scale, with total scores ranging from 26 to 104, reflecting low to high tendency of internet addiction. It has been shown that the CIAS-R has high internal consistency (Cronbach’s α = 0.79–0.93; Chen et al., 2003) and high diagnostic accuracy (AUC = 89.6%; Ko et al., 2005). In the present study, the CIAS-R total score was utilized as an indicator of participants’ current status of internet addiction.

#### Image Acquisition and Processing

Imaging was performed using the GE MR750 3T scanner (GE Healthcare, Waukesha, WI, USA) in the MRI center of National Cheng Kung University. High resolution anatomical images were acquired using fast-SPGR, consisting of 166 axial slices (*TR* = 7.6 ms, *TE* = 3.3 ms, flip angle 171 = 12°, 224 matrices × 224 matrices, slice thickness = 1 mm). Functional images were acquired using a gradient-echo echo-planar imaging (EPI) pulse sequence (*TR* = 2000 ms, *TE* = 30 ms, flip angle = 77°, 64 matrices × 64 matrices, slice thickness = 4 mm, no gap, voxel size 3.4375 mm × 3.4375 mm × 4 mm, 32 axial slices covering the entire brain).

Participants were told to relax and lie in the scanner with their eyes closed. They were asked not to think about any particular event while scanning. The scanning time for the structural image was approximately 3.6 min. The functional image lasted approximately 8 min, with the first five TRs serving as dummy scans to ensure that the signal has reached a steady state before data are collected; thus a run consists of 240 EPI volume images for analysis.

The data was preprocessed using the Data Processing Assistant for Resting-State fMRI (DPARSF; Yan and Zang, 2010), which is based on functions in MRIcroN (Rorden et al., 2007^1^) as well as Statistical Parametric Mapping software (SPM^2^) and the Resting-State fMRI Data Analysis Toolkit (REST; Song et al., 2011) in Matlab (The MathWorks, Inc., Natick, MA, USA). Functional images undergone slice timing correction, followed by realignment to correct for head motion using six-parameter rigid body transformations. The overall motion, characterized by mean framewise displacement (FD), was not large (mean = 0.05, *SD* = 0.03) and did not correlate with the CIAS-R scores (Spearman’s ρ = -0.28, *p* = 0.055), thus impulsivity is not a confounding factor of internet addiction score and motion (Kong et al., 2014). T1 images were coregistered to functional images. Structural images were segmented into CSF, white matter and gray matter based on tissue probability maps in MNI space, and these calculations were used in the subsequent normalization of T1 and EPI images to MNI space. The data were smoothed in the spatial domain using a Gaussian kernel of 6 mm full width at half maximum (FWHM) and removed of linear trend. Nuisance covariates including global mean signal, white matter signal, and cerebrospinal fluid signal were regressed out. Although whether to perform the global signal regression is still an ongoing controversy (e.g., Saad et al., 2012), we decided to implement this method because it has been suggested to maximize the specificity of functional correlations and improve the correspondence between resting-state correlations and anatomy (Fox et al., 2009; Weissenbacher et al., 2009; Takeuchi et al., 2014). Finally, the images undergone band-pass filtering of 0.01–0.08 Hz.

#### Data Analysis

**FIGURE 1:**
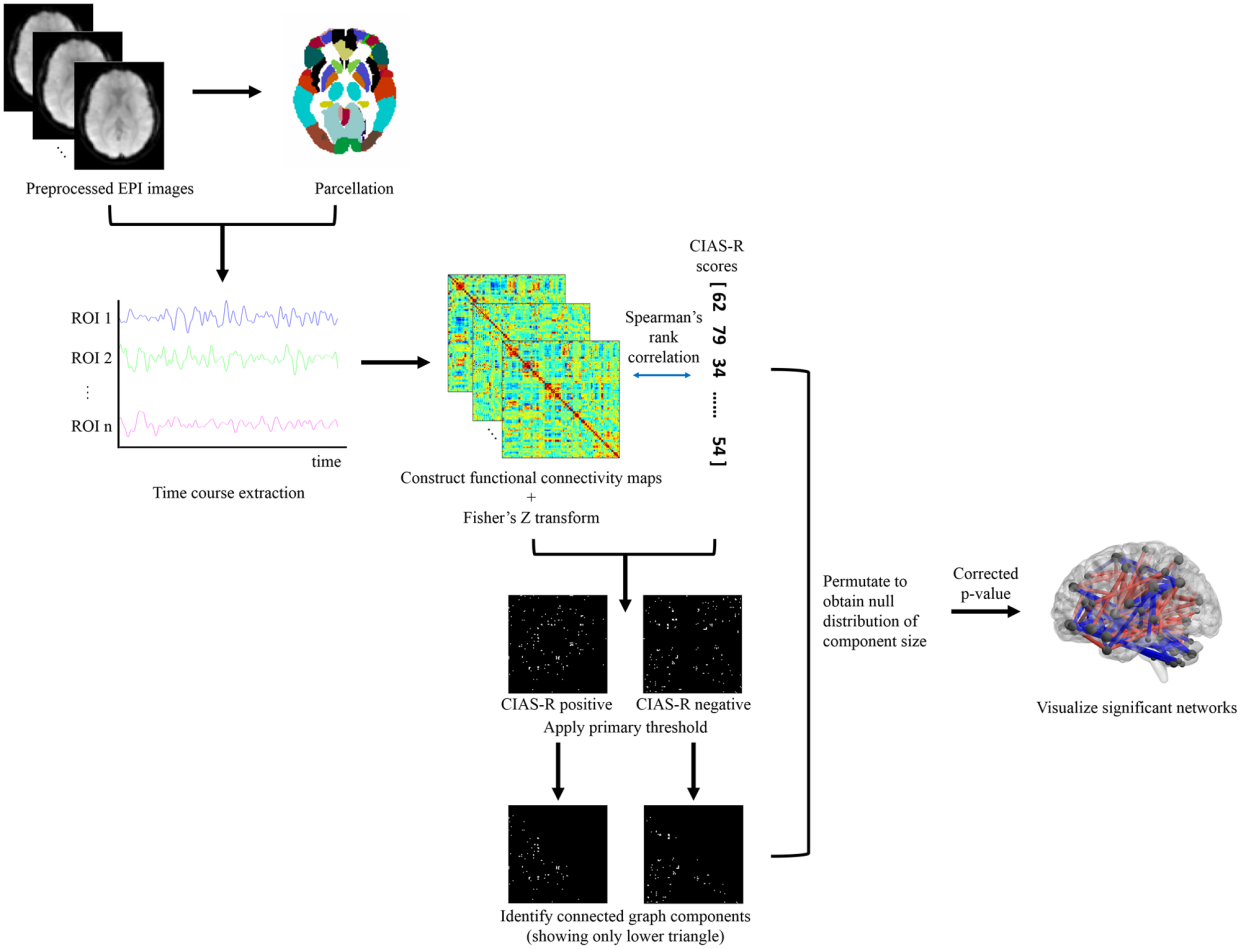
**Flowchart of data analysis pipeline.** Participants’ brains were preprocessed and parcellated to different structural regions according to the AAL template. A correlation matrix was constructed using the time courses extracted from each region to characterize connectivity between each pair of brain region. Network-based statistics was used to identify significant networks related to internet addiction tendency indexed by the CIAS-R score, which included three steps: (1) mass univariate testing of Spearman’s rank correlation of each cell of the correlation matrix and CIAS-R scores, (2) application of primary threshold to select for highly correlated connections, and (3) identify the largest number of connected graph components. The preceding three steps were permutated to obtain a null distribution of the largest component size, and further used to test the significance of the identified network(s).

The fMRI images were parcellated based on the Anatomical Automatic Labeling (AAL; Tzourio-Mazoyer et al., 2002) template, dividing the brain based on anatomical structure into 116 ROIs (or nodes). We chose the AAL atlas because it has been the most commonly used parcellation in functional network studies (Stanley et al., 2013) and was also the template used by Hong et al. (2013), whose study is most relevant to ours, thus increasing the degree of comparability across studies (Zalesky et al., 2010b). The NBS method was used to identify brain networks that consists of inter-regional functional connectivity showing significant correlation with CIAS-R score. The following analyses was done with the aid of the Network Based Statistic Toolbox (Zalesky et al., 2010a) with additional in-house Matlab scripts. A 116 × 116 correlation matrix was constructed for each participant using the time courses extracted from each ROI. The Pearson’s *r* values were normalized to *Z* scores using Fisher’s *Z* transformation. Each cell of the correlation matrix represent the strength of the connection (or edge) between two nodes. Mass univariate testing using Spearman’s rank correlation was performed between participants’ CIAS-R scores and edge strengths within each edge to identify relevant connections that were predictive of the CIAS-R score. Candidate edges that showed high predictability of CIAS-R score were selected via a primary threshold of Spearman’s rho > 0.37 and <-0.37 (approximately the one-tailed alpha = 0.005) respectively, to identify networks that are positively and negatively associated with CIAS-R score. Next, topological clusters, known as connected graph components were identified among the supra-threshold connections. A familywise error (FWE) for the component size was calculated using permutation testing (3000 permutations), which involved randomly reordering the CIAS-R scores and repeating the above process each permutation to obtain a null distribution of the largest component size. Connected graph components whose size exceeds the estimated FWE-corrected *p*-value cutoff of <0.05 were identified as networks that are significantly related to internet addiction tendency. BrainNet Viewer (Xia et al., 2013) was used for the visualization of connections. An illustration of the data analysis pipeline is shown in **Figure 1**.

## Results

### Meta-Analysis

**FIGURE 2:**
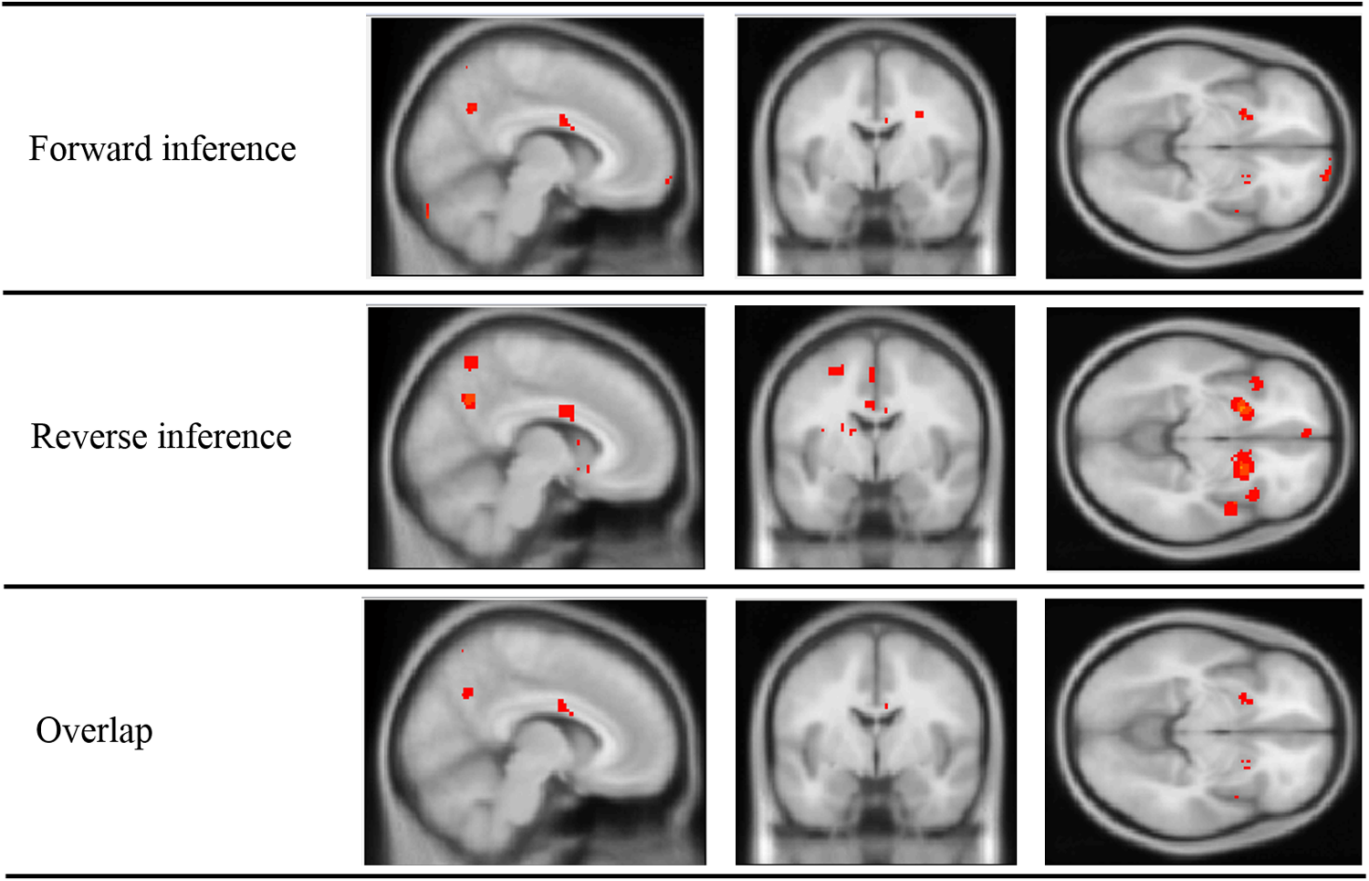
**Inference maps of meta-analysis performed on NeuroSynth, showing regions active in forward inference, reverse inference, and the overlap of the two maps**.

Forward and reverse inference *z*-score maps were generated from NeuroSynth (shown in **Figure 2**). The activations in these two maps show high resemblance of each other. Overlapping these maps revealed activation in regions of the cerebellum, temporal lobe (bilateral inferior temporal gyri, right superior temporal pole, and right middle and superior temporal gyrus), several frontal regions (left middle and superior orbital frontal gyrus, right middle frontal gyrus, right inferior frontal operculum, and right precentral gyrus), bilateral putamen, bilateral insula, right middle cingulate, and right precuneus. **Table 2** lists the clusters identified as well as AAL regions belonging to the cluster.

**Table 2:** Overlapping clusters of forward and reverse inference maps.

Regions		# of voxels (2^∗^2^∗^2)	Peak MNI co-ordinates		
Cluster 1		194	-12	-78	-46
	Cerebelum_Crus2_L				
	Cerebelum_7b_L				
	Cerebelum_Crus1_L				
	Vermis_8				
	Cerebelum_8_L				
	Vermis_7				
Cluster 2		70	-22	-78	-52
	Cerebelum_Crus2_L				
	Cerebelum_7b_L				
	Cerebelum_8_L				
Cluster 3		36	10	0	24
	Cingulum_Mid_R				
Cluster 4		30	-22	52	-18
	Frontal_Mid_Orb_L				
	Frontal_Sup_Orb_L				
Cluster 5		26	10	-62	34
	Precuneus_R				
Cluster 6		20	52	-50	-26
	Temporal_Inf_R				
Cluster 7		19	-24	8	-10
	Putamen_L				
Cluster 8		17	20	10	-10
	Putamen_R				
Cluster 9		17	38	2	34
	Frontal_Mid_R				
	Precentral_R				
	Frontal_Inf_Oper_R				
Cluster 10		14	-30	0	14
	Insula_L				
Cluster 11		13	52	0	-14
	Temporal_Pole_Sup_R			
	Temporal_Sup_R				
Cluster 12		12	46	2	-22
	Temporal_Pole_Sup_R			
	Temporal_Mid_R				
Cluster 13		11	-32	-40	32
	White matter				
Cluster 14		9	26	-38	52
	Postcentral_R				
Cluster 15		8	44	6	-8
	Insula_R				
Cluster 16		6	-44	-32	-22
	Temporal_Inf_L				
Cluster 17		6	6	-64	62
	Precuneus_R				


*R and L stand for right and left. Mid, middle; Sup, superior; Inf, inferior; Orb, orbital; Oper, operculum; Cerebulum, cerebellum.

### Resting-State fMRI

#### Functional Connections Related to Internet Addiction Tendency

**FIGURE 3:**
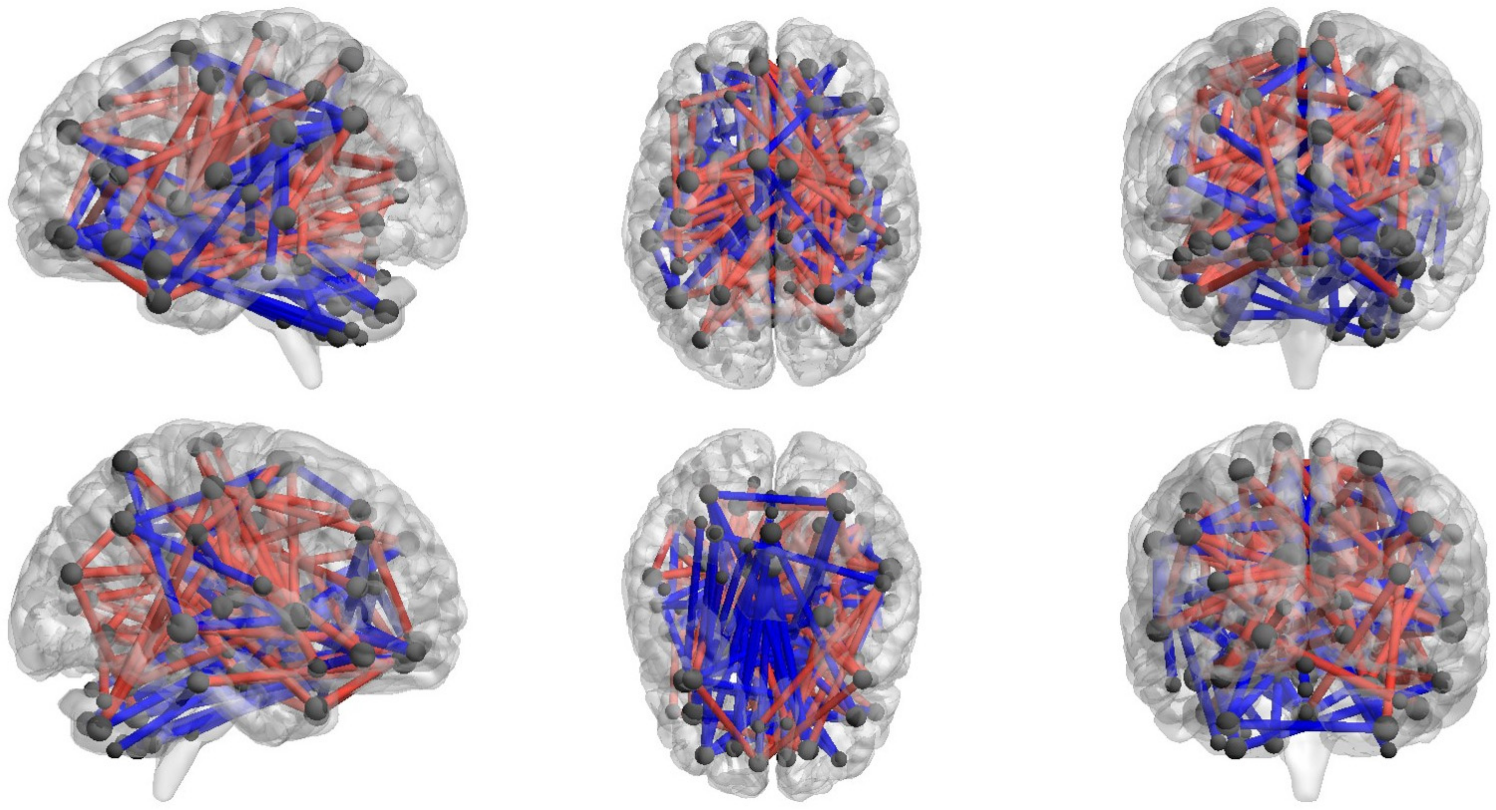
**Network of connections that are correlated with CIAS-R scores.** Gray spheres represent the centroid of each node and are scaled accordingly to the number of significant edges they are associated with. Only nodes with connections are shown. Red lines represent edges that are positively associated with CIAS-R scores, while the blue lines represent edges that are negatively associated with CIAS-R scores.

Using NBS, we identified two networks that showed significant correlation of edge strength and CIAS-R scores (*p* < 0.05, FWE-corrected): one with edges positively correlated with CIAS-R scores (“CIAS-R positive,” shown in red), and one with edges negatively correlated with CIAS-R (“CIAS-R negative,” shown in blue). The CIAS-R positive network consists a total of 65 nodes and 90 edges (45 intrahemispheric, 42 interhemispheric, and 3 connecting to the vermis), while the negative network consist of 64 nodes and 89 edges (35 intrahemispheric, 40 interhemispheric, and 14 connecting to/within the vermis). It is important to note that the two networks are not completely separate, and they share a total of 39 nodes, 30.77% of which are frontal lobe regions. The total number of edges related to CIAS-R consist of 2.68% of all edges of the brain. The network is illustrated in **Figure 3** and specific connections are listed in Supplementary Materials 2, Table S1.

#### Global Distribution of Involved Edges

**FIGURE 4:**
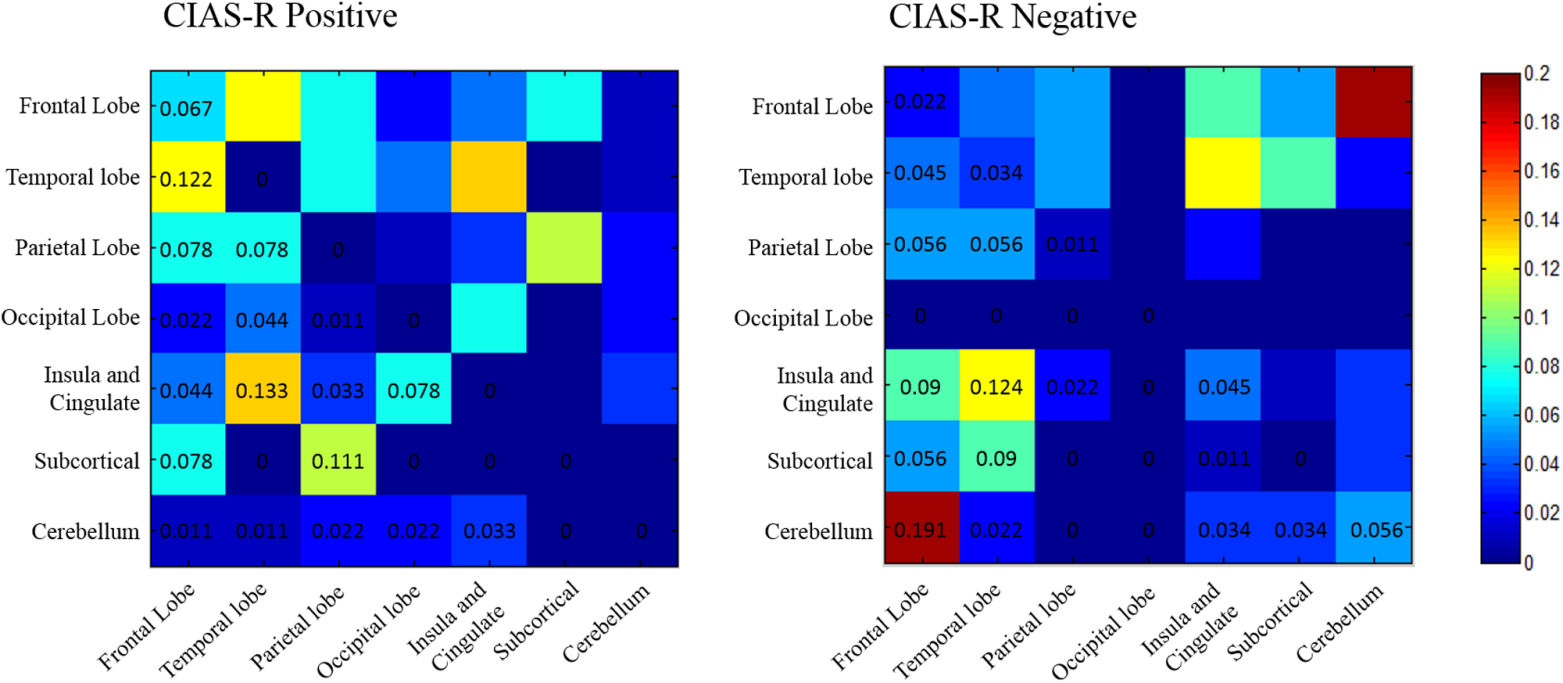
**Proportion of edges that are positively and negatively correlated with internet addiction tendency among pairs of regional subgroupings.** The proportions were calculated by dividing the number of edges between (or within) pairs of regions with the total number of edges identified in each network.

To get a better understanding of how these connections are distributed, we followed Fornito et al. (2011) and Hong et al. (2013), and categorized each AAL region within each network as belonging to seven regional subgroupings: frontal, temporal, parietal, occipital, insula and cingulate gyri, subcortical, and cerebellum. The majority of edges in the CIAS-R positive network involved connections between (1) temporal regions and insula and cingulate gryi (∼13%), most of which involves the posterior cingulate gyrus connecting to various temporal regions; (2) frontal and temporal regions (∼12%), which includes connections between the medial orbitofrontal cortex, paracentral lobule and the temporal lobe gyri, temporal pole; and (3) parietal and subcortical regions (∼11%), consisting of connections between the postcentral cortex and superior parietal lobule with the putamen and pallidum. It is interesting to note that except for the frontal lobe, all others regions do not have any intra-regional connections whose strength is positively correlated with internet addiction tendency. The majority of edges in the CIAS-R negative network involved connections between (1) the frontal lobe and cerebellum (∼19%), most of which are connections between the orbital frontal regions and various ROIs of the cerebellum; and (2) insula and cingulate gyri and the temporal lobe (∼12%), which comprises connections between the insula, cingulum, parahippocampal, and temporal lobe gyri. No occipital regions were found to be included in the CIAS-R negative network. The proportions of inter-regional connections of each network is illustrated in **Figure 4**.

#### Maximally Affected Nodes

**FIGURE 5:**
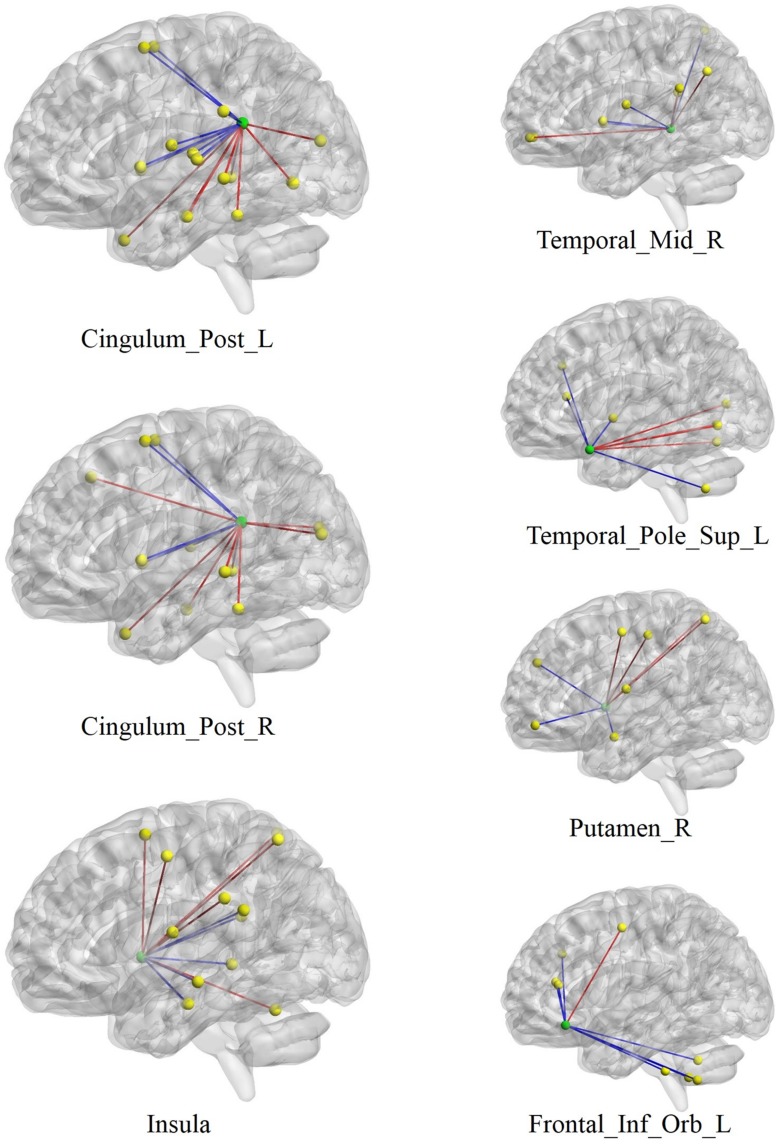
**Visualization of the nodes with highest number of edges related to internet addiction tendency.** Green spheres depict the centroid of each node with maximal edges, while yellow spheres depict their functional connectivity partners. Red lines indicate edges that are positively associated with CIAS-R scores, and blue lines represent edges that are negatively associated with CIAS-R scores. R and L stand for right and left. Post, posterior; Mid, middle; Sup, superior; Inf, inferior; Orb, orbital.

Due to the large number of edges identified, we followed Finn et al. (2014), and identified nodes that have a high “sum of CIAS-R-correlated edges” in order to focus our analysis on regions where connections are maximally related to internet addiction tendency. The sum of CIAS-R-correlated edges of a node was defined as the total number of its edges in both CIAS-R positive and CIAS-R negative networks (this is conceptually equivalent to the degree measure in graph theory). This method will enable us to identify nodes where connections are most likely to be altered by internet addiction tendency. The following **Table 3** lists the nodes that are maximally affected, and shows nodes that have at least a sum of CIAS-R-correlated edges of at least 8. Visualization of the nodes and their connections is displayed in **Figure 5**. These are also the nodes selected for discussion.

**Table 3:** Node level analysis of internet addiction tendency.

AAL Node	MNI coordinate			CIAS-R positive	CIAS-R negative	Sum of correlated edges
Cingulum_Post_L	-4.85	-42.92	24.67	9	8	17
Cingulum_Post_R	7.44	-41.81	21.87	8	5	13
Insula_R	39.02	6.25	2.08	7	6	13
Temporal_Mid_R	57.47	-37.23	-1.47	5	4	9
Temporal_Pole_Sup_L	-39.88	15.14	-20.18	4	5	9
Putamen_R	27.78	4.91	2.46	5	3	8
Frontal_Inf_Orb_L	-35.98	30.71	-12.11	1	7	8


*R and L stand for right and left. Post, posterior; Mid, middle; Sup, superior; Inf, inferior; Orb, orbital.

## Discussion

In a normal group of young adults, we assessed their level of internet addiction through a self-rated questionnaire (CIAS-R), and further identified two brain networks of which functional connections correlated positively and negatively with internet addiction tendency. In the following, we discuss our results at different scales of observation: (1) the crucial regions linking CIAS-R positive and CIAS-R negative networks, (2) regions with high proportions of connections related to internet addiction tendency, and (3) the critical nodes altered by internet addiction tendency.

### Frontal Regions Link CIAS-R Positive and CIAS-R Negative Networks

We observed that the majority of nodes that link the two (CIAS-R positive and CIAS-R negative) networks are located within the frontal lobe. These regions include the superior frontal gyrus, IFG, medial frontal gyrus, rolandic operculum, and supplementary motor area. The prefrontal cortex has been implicated to be a critical structure in cognitive control, inhibition, and response selection (Aron et al., 2004; Talati and Hirsch, 2005; Forstmann et al., 2008). Internet addiction is a phenomenon in that addicts have decreased self-control and decision making regarding internet usage, reflected by continued overuse despite their knowledge of negative effects. For example, several studies have found that participants with internet addiction showed higher fronto-striatal and fronto-parietal activation during the Go/Nogo task (Ding et al., 2014; Ko et al., 2014; Chen et al., 2015) and Stroop task (Dong et al., 2012a, 2013, 2014), suggesting poorer response inhibition and error monitoring, and increased impulsivity. But on the other hand, internet addicts and video game players often show excellent performance of cognitive function, such as motor control and efficient decision making during gaming. Indeed, practice effects of video game play have been shown to generalize to a variety of enhanced executive skills, including perceptual, motor, attentional, and probabilistic inference skills (Green and Bavelier, 2003; Castel et al., 2005; Dye et al., 2009; Green et al., 2010; Green et al., 2012). One fMRI study found reduced recruitment of the fronto-parietal network in video game players compared to non-gamers during a high attentional demand task, possibly reflecting more efficient executive and attentional control (Bavelier et al., 2012). The two faces of cognitive control displayed by internet addicts poses an interesting dilemma. In our study, the observation of frontal regions linking the two networks where functional connectivity is decreased and increased by internet addiction tendency could reflect alterations in the frontal region for different aspects of cognitive control (i.e., for control of internet usage and gaming skills). It is worth mentioning that although Hong et al. (2013) hypothesized there could possibly be increased functional connectivity associated with practice effects in internet addicts, only decreased functional connectivity was observed in their study. One possibility proposed by Hong et al. (2013) for their absence of increased functional connectivity in internet-addicted individuals was that their small sample size resulted in the lack of power. By using seed-based analysis, which requires less multiple comparisons than whole-brain approaches, Hong et al. (2015) re-analyzed the 2013 data and observed both increased and decreased functional connectivity associated with internet addiction.

### The Widely Distributed Connections of the Internet Addiction Tendency Networks

**FIGURE 6:**
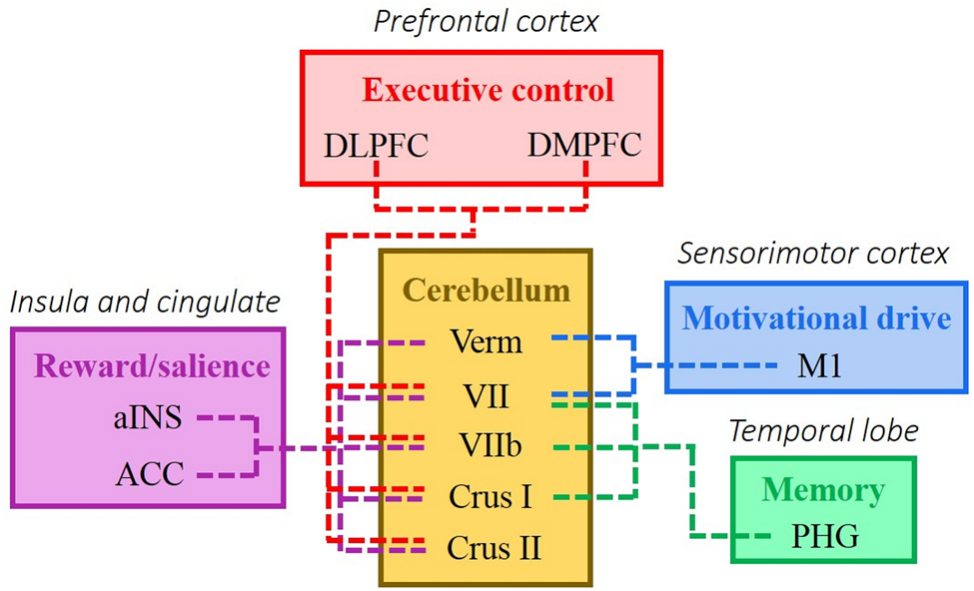
**A model of addiction highlighting the modulating role of the cerebellum of the four major brain networks proposed to be affected by addiction (adapted from Moulton et al., 2014).** These circuits include reward/saliency, motivation/drive, learning/memory, and cognitive control. The colors correspond to different cerebral resting state networks: red, frontoparietal control network and default network; blue, early sensory cortices; green, dorsal attention; and purple, late motor cortices (Buckner et al., 2011; Yeo et al., 2011). ACC, anterior cingulate cortex; aINS, anterior insula; DLPFC, dorsolateral prefrontal cortex; DMPFC, dorsomedial prefrontal cortex; Hypo, hypothalamus; M1, primary motor cortex; PHG, parahippocampal gyrus; sACC, subgenual anterior cingulate cortex; Verm, vermis; VI, cerebellar hemispheric lobule VI; VIIb, cerebellar hemispheric lobule VIIb.

The data shows a large number of inter- and intra-hemispheric connections in both CIAS-R positive and CIAS-R negative networks, reflecting the extensive influence of internet addiction tendency on the brain. We observed that the highest proportion of connections in the CIAS-R positive network involved the “insula and cingulate – temporal,” “frontal – temporal,” and “subcortical – parietal” edges, while the highest proportion of connections in the CIAS-R negative network involved “frontal – cerebellar” and “insula and cingulate – temporal” edges (**Figure 4**). In a recently proposed model of addiction (Moulton et al., 2014), the cerebellum helps maintain the homeostasis of the four interconnected circuits relevant to addiction: reward/saliency, motivation/drive, learning/memory as well as cognitive control. This model integrates the four-circuit model (Volkow et al., 2003, 2010) and the cerebellar functional resting state networks relating to executive and associative processing in the cerebral cortex (Buckner et al., 2011). The components for reward/saliency, motivation/drive, and learning/memory are amplified, while cognitive control is diminished in addiction. See **Figure 6** for an illustration. Our observations of the highest functional connectivity proportions of the two internet addiction tendency networks are generally compatible with Moulton et al.’s (2014) model of the critical components involved in the addiction circuitry. Likewise, we did not observe many significant connections comprising the occipital lobe, which also dovetails Hong et al.’s (2013) findings. However, we additionally found a great proportion of “subcortical – parietal” edges that although is not particularly highlighted in the four-circuit model, these connections have been observed in the internet addiction literature (e.g., Ding et al., 2013; Hong et al., 2013, 2015), which could be due to a practice effect relating to internet usage.

### Critical Nodes Altered by Internet Addiction Tendency

We identified nodes with the most connections are maximally related to internet addiction tendency. These nodes are those whose pattern of connections between the node itself and other brain regions are most susceptible to alteration by internet addiction tendency. The regions are specifically the bilateral posterior cingulate gyrus, right insula, right middle temporal gyrus, left superior temporal pole, right putamen, and the orbital part of left IFG (**Figure 5**). These regions have been implicated as key regions in many (internet) addiction studies and some have already been mentioned in the previous section. We now discuss the addiction literature highlighting these regions in more detail. The PCC, part of the default mode network and involved in various aspects of self-processing (Buckner et al., 2008; Fransson and Marrelec, 2008), served as a seed region in Ding et al.’s (2013) study, which showed significantly increased functional connectivity with the bilateral cerebellum posterior lobe and middle temporal gyrus, while decreased bilateral inferior parietal lobule and right inferior temporal gyrus in internet gaming addicts. Internet addicts have also been found to show abnormal fractional anisotropy (Dong et al., 2012b) and gray matter density (Zhou et al., 2011) in the PCC. Zhang et al. (2015) chose the insula, which has been implicated in addiction (Naqvi and Bechara, 2009; Droutman et al., 2015), as the seed region and found altered functional connectivity with a network of regions in internet addicts. The role of the insula in addiction has been suggested for integrating interoceptive signals into conscious feelings (drug urges) and biases behavior during decision making (Naqvi and Bechara, 2009). The middle temporal gyrus and superior temporal pole has been observed in some internet addiction studies (see Meng et al., 2015 for a meta-analysis), and have been associated with game urge/craving, semantic processing, disembodiment, working memory, and emotional processing; however, their specific roles in addiction require further investigations. The putamen, part of the dorsal striatum, is also a critical region suggested by many addiction research (e.g., Ko et al., 2009; Ding et al., 2013; Lin et al., 2015), in which concomitant dopamine neurotransmission is involved in the development of compulsive drug-seeking and craving (Volkow et al., 2006; Koob and Volkow, 2010). Furthermore, research has suggested that dysfunction with the striato-thalamo-orbitofrontal circuit is a crucial cause of addiction, while the dorsal striatum involved in habit-learning and craving, the orbitofrontal cortex is involved with salience, drive, and compulsivity (Volkow and Fowler, 2000; Koob and Volkow, 2010; Volkow et al., 2010; Goldstein and Volkow, 2011). The abnormal functioning of the orbitofrontal cortex could explain the behavioral malfunctioning in addiction. Summarizing the above, the nodes we identified are hubs that are most susceptible to alteration by internet addiction tendency, and they have been identified repeatedly in the existing literature.

### Limitation

As pointed out by one of our reviewers, whether to perform global signal regression in resting-state fMRI still remains a current debate. After re-analyzing the current data without global signal regression, our results turned out quite different compared to our original analysis and only 22.91% of the edges found in the NBS analyses without global signal regression overlapped with those of our current results. Without global signal regression, we did not find sufficient functional connections that were positively related to CIAS-R scores; however, we did find a network that comprised of functional connections that were negatively related to CIAS-R scores. When identifying nodes with the most connections are maximally related to internet addiction tendency, we find consistency with the global signal regression analysis in that the cingulate, insula, temporal, and frontal areas were the most involved. However, several differences include the additional finding of bilateral supplementary motor areas and right angular gyrus showing decreased functional connectivity, and there were not as many subcortical regions in the identified network. While global signal regression still remains controversial, we decided to report both results. Details of the network identified without global signal regression is documented in the Supplementary Materials 3. Hopefully, future work on image preprocessing will shed light on which result is more accurate. At this moment, we suggest to interpret the current results with such caveats in mind.

## Conclusion

Using a data-driven approach, we showed that network based statistics is a useful tool to characterize whole-brain connectivity affected by internet addiction tendency, identifying connections and critical regions that echo previous studies. Compared to seed analyses, this whole-brain approach provides a more comprehensive analysis of brain connections related to internet addiction, investigating a total of 6670 connections. We further showed that many functional connections and brain regions critical in clinical cases of addiction are also found to be associated with pre-clinical tendencies indexed by behavioral questionnaire measures. Although using a correlational approach, we cannot be sure whether these networks are altered as a result of internet usage or whether they are characteristics of people who are predisposed to higher risk of developing internet addiction, this research provides useful information in helping us understand the neural characteristics underlying addiction and its development.

## Author Contributions

TW performed the experiment, analyzed the data, interpreted the results, wrote and revised the manuscript. SH designed the experiment, wrote the grant proposal, guided the experiment’s preparation and execution, helped in interpreting the data, preparing, and revising the manuscript.

## Conflict of Interest Statement

The authors declare that the research was conducted in the absence of any commercial or financial relationships that could be construed as a potential conflict of interest.

## Footnotes

1 http://www.mricro.com

2 http://www.fil.ion.ucl.ac.uk/spm
