# Supplementary material for: Network-Based Analysis Reveals Functional Connectivity Related to Internet Addiction Tendency
Source: Front Hum Neurosci. 2016 Feb 1;10:6. doi: 10.3389/fnhum.2016.00006 (PMC4740778; doi:10.3389/fnhum.2016.00006)
Supplement: Supplementary file 1 [file Data_Sheet_1.DOCX]

Bavelier, D., Achtman, R. L., Mani, M., & Föcker, J. (2012). Neural bases of selective attention in action video game players. *Vision research*, *61*, 132-143.

Cole SW, Yoo DJ, Knutson B (2012) Interactivity and reward-related neural activation during a serious videogame. *PloS one* 7(3): e33909. doi:10.1371/journal.pone.0033909

Ding, W. N., Sun, J. H., Sun, Y. W., Zhou, Y., Li, L., Xu, J. R., & Du, Y. S. (2013). Altered default network resting-state functional connectivity in adolescents with Internet gaming addiction. *PloS one*, *8*(3).

Dong, G., DeVito, E. E., Du, X., & Cui, Z. (2012). Impaired inhibitory control in ‘internet addiction disorder’: a functional magnetic resonance imaging study. *Psychiatry Research: Neuroimaging*, *203*(2), 153-158.

Dong, G., Hu, Y., Lin, X., & Lu, Q. (2013). What makes Internet addicts continue playing online even when faced by severe negative consequences? Possible explanations from an fMRI study. *Biological psychology*, *94*(2), 282-289.

Granek, J. A., Gorbet, D. J., & Sergio, L. E. (2010). Extensive video-game experience alters cortical networks for complex visuomotor transformations. *Cortex*, *46*(9), 1165-1177.

Han, D. H., Kim, S. M., Lee, Y. S., & Renshaw, P. F. (2012). The effect of family therapy on the changes in the severity of on-line game play and brain activity in adolescents with on-line game addiction. *Psychiatry Research: Neuroimaging*, *202*(2), 126-131.

Hong, S. B., Harrison, B. J., Dandash, O., Choi, E. J., Kim, S. C., Kim, H. H., ... & Yi, S. H. (2015). A selective involvement of putamen functional connectivity in youth with internet gaming disorder. *Brain research*, *1602*, 85-95.

Kätsyri, J., Hari, R., Ravaja, N., & Nummenmaa, L. (2013). The opponent matters: elevated fMRI reward responses to winning against a human versus a computer opponent during interactive video game playing. *Cerebral Cortex*,*23*(12), 2829-2839.

Kätsyri, J., Hari, R., Ravaja, N., & Nummenmaa, L. (2013). Just watching the game ain't enough: striatal fMRI reward responses to successes and failures in a video game during active and vicarious playing. *Frontiers in human neuroscience*, *7*.

Klasen, M., Weber, R., Kircher, T. T., Mathiak, K. A., & Mathiak, K. (2012). Neural contributions to flow experience during video game playing. *Social cognitive and affective neuroscience*, *7*(4), 485-495.

Ko, C. H., Hsieh, T. J., Chen, C. Y., Yen, C. F., Chen, C. S., Yen, J. Y., ... & Liu, G. C. (2014). Altered brain activation during response inhibition and error processing in subjects with Internet gaming disorder: a functional magnetic imaging study. *European archives of psychiatry and clinical neuroscience*, *264*(8), 661-672.

Liu, J., Li, W., Zhou, S., Zhang, L., Wang, Z., Zhang, Y., ... & Li, L. (2015). Functional characteristics of the brain in college students with internet gaming disorder. *Brain imaging and behavior*, 1-8.

Lorenz, R. C., Gleich, T., Gallinat, J., & Kühn, S. (2015). Video game training and the reward system. *Frontiers in human neuroscience*, *9*.

Luijten, M., Meerkerk, G. J., Franken, I. H., van de Wetering, B. J., & Schoenmakers, T. M. (2015). An fMRI study of cognitive control in problem gamers. *Psychiatry Research: Neuroimaging*, *231*(3), 262-268.

Mathiak, K., & Weber, R. (2006). Toward brain correlates of natural behavior: fMRI during violent video games. *Human brain mapping*, *27*(12), 948-956.

Sun, Y., Ying, H., Seetohul, R. M., Xuemei, W., Ya, Z., Qian, L., ... & Ye, S. (2012). Brain fMRI study of crave induced by cue pictures in online game addicts (male adolescents). *Behavioural brain research*, *233*(2), 563-576.

Wee C-Y, Zhao Z, Yap P-T, Wu G, Shi F, Price T, et al. (2014) Disrupted brain functional network in internet addiction disorder: a resting-state functional magnetic resonance imaging study. *PloS one 9*(9): e107306. doi:10.1371/journal.pone.0107306
