# Supplementary material for: Network-Based Analysis Reveals Functional Connectivity Related to Internet Addiction Tendency
Source: Front Hum Neurosci. 2016 Feb 1;10:6. doi: 10.3389/fnhum.2016.00006 (PMC4740778; doi:10.3389/fnhum.2016.00006)
Supplement: Supplementary file 2 [file Data_Sheet_2.DOCX]

**Supplementary Table 1A:** List of connections within the CIAS-R positive network.

| CIAS-R positive | |  |  |  |  |
| --- | --- | --- | --- | --- | --- |
| Node1 | Node2 | Region1 | Region2 | rho | p-value |
| Precentral_L | Frontal_Inf_Orb_L | F | F | 0.406015 | 0.002316 |
| Precentral_L | Frontal_Inf_Orb_R | F | F | 0.383227 | 0.00392 |
| Precentral_L | Insula_R | F | I | 0.404743 | 0.002387 |
| Precentral_L | Putamen_L | F | S | 0.450492 | 0.000743 |
| Precentral_L | Putamen_R | F | S | 0.423193 | 0.001519 |
| Precentral_L | Pallidum_L | F | S | 0.498438 | 0.000181 |
| Precentral_L | Pallidum_R | F | S | 0.526605 | 7.17E-05 |
| Precentral_R | Supp_Motor_Area_R | F | F | 0.417525 | 0.00175 |
| Precentral_R | Cerebelum_Crus2_R | F | C | 0.39867 | 0.002755 |
| Frontal_Sup_L | Postcentral_R | F | P | 0.374031 | 0.0048 |
| Frontal_Sup_R | Frontal_Med_Orb_R | F | F | 0.408213 | 0.002197 |
| Frontal_Sup_R | Cingulum_Post_R | F | I | 0.393638 | 0.003096 |
| Frontal_Sup_Orb_R | Frontal_Inf_Tri_L | F | F | 0.410584 | 0.002074 |
| Frontal_Sup_Orb_R | Temporal_Inf_R | F | T | 0.381781 | 0.004048 |
| Frontal_Inf_Tri_L | Parietal_Inf_L | F | P | 0.374263 | 0.004776 |
| Frontal_Inf_Tri_R | Parietal_Inf_L | F | P | 0.377039 | 0.004495 |
| Frontal_Inf_Orb_R | Parietal_Sup_L | F | P | 0.386698 | 0.003626 |
| Frontal_Inf_Orb_R | Parietal_Inf_L | F | P | 0.391324 | 0.003265 |
| Rolandic_Oper_L | Insula_R | F | I | 0.404627 | 0.002393 |
| Rolandic_Oper_L | Putamen_L | F | S | 0.404106 | 0.002423 |
| Rolandic_Oper_L | Putamen_R | F | S | 0.460671 | 0.00056 |
| Rolandic_Oper_R | Heschl_R | F | T | 0.370561 | 0.005173 |
| Supp_Motor_Area_L | Supp_Motor_Area_R | F | F | 0.421631 | 0.00158 |
| Supp_Motor_Area_L | Insula_R | F | I | 0.397976 | 0.0028 |
| Supp_Motor_Area_R | SupraMarginal_R | F | P | 0.383632 | 0.003885 |
| Frontal_Sup_Medial_L | Calcarine_R | F | O | 0.373569 | 0.004848 |
| Frontal_Sup_Medial_L | Postcentral_R | F | P | 0.410411 | 0.002083 |
| Frontal_Med_Orb_L | Temporal_Mid_R | F | T | 0.410237 | 0.002092 |
| Frontal_Med_Orb_L | Temporal_Pole_Mid_L | F | T | 0.380856 | 0.004132 |
| Frontal_Med_Orb_L | Temporal_Pole_Mid_R | F | T | 0.426431 | 0.0014 |
| Frontal_Med_Orb_R | ParaHippocampal_R | F | T | 0.426779 | 0.001388 |
| Frontal_Med_Orb_R | Temporal_Mid_R | F | T | 0.39092 | 0.003295 |
| Rectus_L | Temporal_Inf_R | F | T | 0.378774 | 0.004327 |
| Rectus_R | Occipital_Mid_L | F | O | 0.376171 | 0.004581 |
| Insula_L | Vermis_6 | I | C | 0.382186 | 0.004012 |
| Insula_R | Parietal_Sup_L | I | P | 0.45749 | 0.000612 |
| Insula_R | Parietal_Sup_R | I | P | 0.371891 | 0.005027 |
| Insula_R | SupraMarginal_L | I | P | 0.403528 | 0.002457 |
| Insula_R | Cerebelum_6_R | I | C | 0.37033 | 0.005199 |
| Cingulum_Ant_L | Lingual_L | I | O | 0.400058 | 0.002666 |
| Cingulum_Ant_L | Fusiform_L | I | T | 0.431232 | 0.001238 |
| Cingulum_Ant_R | Lingual_L | I | O | 0.374725 | 0.004728 |
| Cingulum_Mid_R | Cerebelum_6_R | I | C | 0.414691 | 0.001877 |
| Cingulum_Post_L | ParaHippocampal_L | I | T | 0.379699 | 0.004239 |
| Cingulum_Post_L | ParaHippocampal_R | I | T | 0.45882 | 0.00059 |
| Cingulum_Post_L | Lingual_L | I | O | 0.372875 | 0.004922 |
| Cingulum_Post_L | Lingual_R | I | O | 0.41417 | 0.001901 |
| Cingulum_Post_L | Occipital_Mid_L | I | O | 0.461943 | 0.00054 |
| Cingulum_Post_L | Fusiform_L | I | T | 0.459688 | 0.000576 |
| Cingulum_Post_L | Temporal_Mid_L | I | T | 0.419491 | 0.001667 |
| Cingulum_Post_L | Temporal_Mid_R | I | T | 0.564083 | 1.82E-05 |
| Cingulum_Post_L | Temporal_Pole_Mid_R | I | T | 0.398496 | 0.002766 |
| Cingulum_Post_R | ParaHippocampal_R | I | T | 0.391729 | 0.003234 |
| Cingulum_Post_R | Occipital_Mid_L | I | O | 0.407982 | 0.002209 |
| Cingulum_Post_R | Occipital_Mid_R | I | O | 0.407172 | 0.002252 |
| Cingulum_Post_R | Fusiform_L | I | T | 0.411683 | 0.00202 |
| Cingulum_Post_R | Temporal_Mid_L | I | T | 0.381434 | 0.00408 |
| Cingulum_Post_R | Temporal_Mid_R | I | T | 0.565009 | 1.76E-05 |
| Cingulum_Post_R | Temporal_Pole_Mid_R | I | T | 0.379584 | 0.00425 |
| ParaHippocampal_L | Angular_R | T | P | 0.426258 | 0.001406 |
| ParaHippocampal_R | Angular_R | T | P | 0.439907 | 0.000987 |
| Amygdala_R | Paracentral_Lobule_R | T | F | 0.387912 | 0.003528 |
| Calcarine_L | Temporal_Pole_Mid_L | O | T | 0.414401 | 0.00189 |
| Calcarine_R | Temporal_Pole_Sup_L | O | T | 0.416831 | 0.001781 |
| Lingual_L | Temporal_Pole_Sup_L | O | T | 0.398323 | 0.002777 |
| Lingual_L | Cerebelum_Crus1_R | O | C | 0.389821 | 0.003379 |
| Lingual_R | Temporal_Pole_Sup_L | O | T | 0.406709 | 0.002277 |
| Occipital_Mid_R | Precuneus_R | O | P | 0.432331 | 0.001203 |
| Occipital_Mid_R | Vermis_9 | O | C | 0.396645 | 0.002888 |
| Postcentral_L | Putamen_L | P | S | 0.384789 | 0.003785 |
| Postcentral_L | Putamen_R | P | S | 0.387392 | 0.00357 |
| Postcentral_L | Pallidum_L | P | S | 0.390226 | 0.003348 |
| Postcentral_L | Pallidum_R | P | S | 0.401041 | 0.002606 |
| Postcentral_R | Heschl_R | P | T | 0.380509 | 0.004164 |
| Postcentral_R | Cerebelum_Crus2_R | P | C | 0.44841 | 0.000786 |
| Parietal_Sup_L | Putamen_R | P | S | 0.514575 | 0.000108 |
| Parietal_Sup_L | Pallidum_L | P | S | 0.471718 | 0.000408 |
| Parietal_Sup_L | Pallidum_R | P | S | 0.481261 | 0.000308 |
| Parietal_Sup_R | Putamen_L | P | S | 0.38103 | 0.004116 |
| Parietal_Sup_R | Putamen_R | P | S | 0.440081 | 0.000983 |
| Parietal_Sup_R | Pallidum_R | P | S | 0.410642 | 0.002072 |
| SupraMarginal_L | Temporal_Sup_R | P | T | 0.441296 | 0.000951 |
| SupraMarginal_R | Cerebelum_6_R | P | C | 0.380914 | 0.004127 |
| Angular_L | Temporal_Mid_L | P | T | 0.444592 | 0.000871 |
| Angular_L | Temporal_Mid_R | P | T | 0.478543 | 0.000334 |
| Angular_L | Temporal_Pole_Mid_R | P | T | 0.373048 | 0.004903 |
| Paracentral_Lobule_L | Putamen_L | F | S | 0.386582 | 0.003636 |
| Paracentral_Lobule_L | Temporal_Pole_Sup_R | F | T | 0.38502 | 0.003766 |
| Paracentral_Lobule_R | Temporal_Pole_Sup_R | F | T | 0.480683 | 0.000313 |
| Temporal_Pole_Sup_L | Vermis_6 | T | C | 0.377501 | 0.00445 |
| *F: frontal lobe; T: temporal lobe; P: parietal lobe; O: occipital lobe; I: insula and cingulate cortex; S: subcortical; C: cerebellum | | | | | |
| **Sup: superior; Orb: orbital; Mid: middle; Inf: inferior; Oper: opercular/operculum; Tri: triangular; Supp: supplementary; Med: medial; Ant: anterior; Post: posterior; Cerebelum: cerebellum | | | | | |

**Supplementary Table 1B:** List of connections within the CIAS-R negative network.

| CIAS-R negative | |  |  |  |  |
| --- | --- | --- | --- | --- | --- |
| Node1 | Node2 | Region1 | Region2 | rho | p-value |
| Frontal_Sup_R | Supp_Motor_Area_L | F | F | -0.37264 | 0.004946 |
| Frontal_Sup_Orb_L | Cerebelum_3_L | F | C | -0.42863 | 0.001324 |
| Frontal_Sup_Orb_L | Vermis_1_2 | F | C | -0.42024 | 0.001636 |
| Frontal_Sup_Orb_L | Vermis_9 | F | C | -0.3716 | 0.005059 |
| Frontal_Sup_Orb_R | Cerebelum_3_L | F | C | -0.44754 | 0.000805 |
| Frontal_Sup_Orb_R | Vermis_1_2 | F | C | -0.4417 | 0.000941 |
| Frontal_Sup_Orb_R | Vermis_3 | F | C | -0.39092 | 0.003295 |
| Frontal_Mid_R | Frontal_Inf_Orb_L | F | F | -0.41984 | 0.001653 |
| Frontal_Mid_R | Temporal_Pole_Sup_L | F | T | -0.44274 | 0.000916 |
| Frontal_Mid_Orb_L | Cingulum_Ant_R | F | I | -0.40422 | 0.002417 |
| Frontal_Mid_Orb_L | Putamen_R | F | S | -0.38577 | 0.003703 |
| Frontal_Mid_Orb_L | Cerebelum_8_L | F | C | -0.37189 | 0.005027 |
| Frontal_Mid_Orb_L | Vermis_8 | F | C | -0.38248 | 0.003986 |
| Frontal_Mid_Orb_R | Caudate_L | F | S | -0.5284 | 6.74E-05 |
| Frontal_Mid_Orb_R | Thalamus_L | F | S | -0.44471 | 0.000869 |
| Frontal_Inf_Tri_R | Temporal_Pole_Sup_L | F | T | -0.40445 | 0.002403 |
| Frontal_Inf_Tri_R | Temporal_Pole_Mid_L | F | T | -0.38895 | 0.003446 |
| Frontal_Inf_Orb_L | Cingulum_Ant_L | F | I | -0.38988 | 0.003374 |
| Frontal_Inf_Orb_L | Cingulum_Ant_R | F | I | -0.42846 | 0.00133 |
| Frontal_Inf_Orb_L | Cerebelum_8_L | F | C | -0.41452 | 0.001885 |
| Frontal_Inf_Orb_L | Cerebelum_9_L | F | C | -0.38976 | 0.003383 |
| Frontal_Inf_Orb_L | Cerebelum_10_L | F | C | -0.37287 | 0.004922 |
| Frontal_Inf_Orb_L | Vermis_9 | F | C | -0.39803 | 0.002796 |
| Frontal_Inf_Orb_R | Thalamus_L | F | S | -0.43441 | 0.00114 |
| Frontal_Inf_Orb_R | Vermis_9 | F | C | -0.39202 | 0.003213 |
| Rolandic_Oper_L | Cingulum_Post_L | F | I | -0.42857 | 0.001326 |
| Rolandic_Oper_L | Angular_L | F | P | -0.43285 | 0.001187 |
| Rolandic_Oper_L | Angular_R | F | P | -0.417 | 0.001773 |
| Rolandic_Oper_L | Temporal_Mid_R | F | T | -0.37392 | 0.004812 |
| Rolandic_Oper_R | Angular_R | F | P | -0.38068 | 0.004148 |
| Supp_Motor_Area_L | Cingulum_Post_L | F | I | -0.4568 | 0.000624 |
| Supp_Motor_Area_L | Cingulum_Post_R | F | I | -0.42452 | 0.001469 |
| Supp_Motor_Area_L | Angular_R | F | P | -0.40977 | 0.002115 |
| Supp_Motor_Area_R | Cingulum_Post_L | F | I | -0.52741 | 6.97E-05 |
| Supp_Motor_Area_R | Cingulum_Post_R | F | I | -0.4871 | 0.000258 |
| Supp_Motor_Area_R | Angular_L | F | P | -0.45581 | 0.000641 |
| Frontal_Sup_Medial_L | Putamen_R | F | S | -0.39231 | 0.003192 |
| Frontal_Sup_Medial_L | Vermis_1_2 | F | C | -0.3941 | 0.003063 |
| Frontal_Sup_Medial_L | Vermis_7 | F | C | -0.44384 | 0.000889 |
| Frontal_Med_Orb_L | Vermis_1_2 | F | C | -0.45813 | 0.000601 |
| Frontal_Med_Orb_R | Vermis_1_2 | F | C | -0.38178 | 0.004048 |
| Insula_L | Cingulum_Post_L | I | I | -0.40393 | 0.002433 |
| Insula_L | Cingulum_Post_R | I | I | -0.43418 | 0.001147 |
| Insula_L | ParaHippocampal_L | I | T | -0.414 | 0.001909 |
| Insula_L | ParaHippocampal_R | I | T | -0.46449 | 0.000503 |
| Insula_L | Temporal_Mid_R | I | T | -0.42666 | 0.001392 |
| Insula_R | Cingulum_Post_L | I | I | -0.46657 | 0.000474 |
| Insula_R | Cingulum_Post_R | I | I | -0.4557 | 0.000644 |
| Insula_R | Hippocampus_L | I | T | -0.42556 | 0.001431 |
| Insula_R | ParaHippocampal_L | I | T | -0.40069 | 0.002627 |
| Insula_R | ParaHippocampal_R | I | T | -0.43806 | 0.001037 |
| Insula_R | Temporal_Mid_R | I | T | -0.47293 | 0.000394 |
| Cingulum_Ant_L | Caudate_R | I | S | -0.43997 | 0.000986 |
| Cingulum_Ant_L | Cerebelum_Crus2_R | I | C | -0.39485 | 0.00301 |
| Cingulum_Ant_L | Cerebelum_7b_R | I | C | -0.38803 | 0.003519 |
| Cingulum_Ant_R | Cerebelum_Crus2_R | I | C | -0.39769 | 0.002819 |
| Cingulum_Mid_R | Angular_L | I | P | -0.46536 | 0.00049 |
| Cingulum_Post_L | SupraMarginal_L | I | P | -0.37363 | 0.004842 |
| Cingulum_Post_L | Heschl_L | I | T | -0.40046 | 0.002641 |
| Cingulum_Post_L | Temporal_Sup_L | I | T | -0.37692 | 0.004506 |
| Cingulum_Post_L | Temporal_Sup_R | I | T | -0.47131 | 0.000413 |
| Cingulum_Post_R | Heschl_R | I | T | -0.41891 | 0.001691 |
| Hippocampus_L | Temporal_Sup_L | T | T | -0.37727 | 0.004472 |
| Hippocampus_L | Temporal_Sup_R | T | T | -0.38404 | 0.00385 |
| Hippocampus_R | ParaHippocampal_L | T | T | -0.38757 | 0.003556 |
| ParaHippocampal_L | Pallidum_L | T | S | -0.40873 | 0.002169 |
| ParaHippocampal_R | Pallidum_L | T | S | -0.38276 | 0.003961 |
| Amygdala_L | Caudate_R | T | S | -0.39693 | 0.002868 |
| Amygdala_L | Putamen_L | T | S | -0.38514 | 0.003756 |
| Amygdala_L | Putamen_R | T | S | -0.41232 | 0.001989 |
| Amygdala_L | Pallidum_L | T | S | -0.37045 | 0.005186 |
| Parietal_Sup_R | Temporal_Mid_R | P | T | -0.37854 | 0.004349 |
| SupraMarginal_L | Angular_L | P | P | -0.37542 | 0.004657 |
| SupraMarginal_L | Temporal_Mid_L | P | T | -0.41833 | 0.001715 |
| SupraMarginal_L | Temporal_Pole_Mid_L | P | T | -0.46466 | 0.0005 |
| SupraMarginal_L | Temporal_Inf_L | P | T | -0.37669 | 0.004529 |
| SupraMarginal_R | Temporal_Pole_Mid_L | P | T | -0.39734 | 0.002841 |
| Caudate_L | Cerebelum_Crus2_L | S | C | -0.51417 | 0.000109 |
| Caudate_R | Cerebelum_Crus2_L | S | C | -0.43586 | 0.001098 |
| Pallidum_L | Temporal_Pole_Sup_L | S | T | -0.41145 | 0.002031 |
| Pallidum_R | Temporal_Pole_Sup_L | S | T | -0.48074 | 0.000313 |
| Thalamus_L | Cerebelum_Crus2_L | S | C | -0.38219 | 0.004012 |
| Temporal_Pole_Sup_L | Cerebelum_7b_L | T | C | -0.38409 | 0.003845 |
| Temporal_Inf_R | Cerebelum_8_L | T | C | -0.37021 | 0.005212 |
| Cerebelum_Crus2_L | Cerebelum_Crus2_R | C | C | -0.46235 | 0.000534 |
| Cerebelum_3_L | Cerebelum_9_L | C | C | -0.39115 | 0.003278 |
| Cerebelum_3_L | Vermis_9 | C | C | -0.37704 | 0.004495 |
| Vermis_1_2 | Vermis_9 | C | C | -0.37409 | 0.004794 |
| Vermis_3 | Vermis_10 | C | C | -0.38965 | 0.003392 |
| *F: frontal lobe; T: temporal lobe; P: parietal lobe; O: occipital lobe; I: insula and cingulate cortex; S: subcortical; C: cerebellum | | | | | |
| **Sup: superior; Orb: orbital; Mid: middle; Inf: inferior; Oper: opercular/operculum; Tri: triangular; Supp: supplementary; Med: medial; Ant: anterior; Post: posterior; Cerebelum: cerebellum | | | | | |
