# Supplementary material for: Network-Based Analysis Reveals Functional Connectivity Related to Internet Addiction Tendency
Source: Front Hum Neurosci. 2016 Feb 1;10:6. doi: 10.3389/fnhum.2016.00006 (PMC4740778; doi:10.3389/fnhum.2016.00006)
Supplement: Supplementary file 3 [file Data_Sheet_3.DOCX]

**Results without global signal regression**

*Functional connections related to internet addiction tendency*

Using NBS, we identified a network that showed significant negative correlation of edge strength and CIAS-R scores (p < .05, FWE-corrected). The CIAS-R positive network consists a total of 76 nodes and 197 edges (109 intrahemispheric, 75 interhemispheric, and 13 connecting to the vermis). The total number of identified edges related to CIAS-R consist of 2.95% of all edges of the brain. The specific connections illustrated in Supplementary Figure 1 and listed in Supplementary Table 2 below.


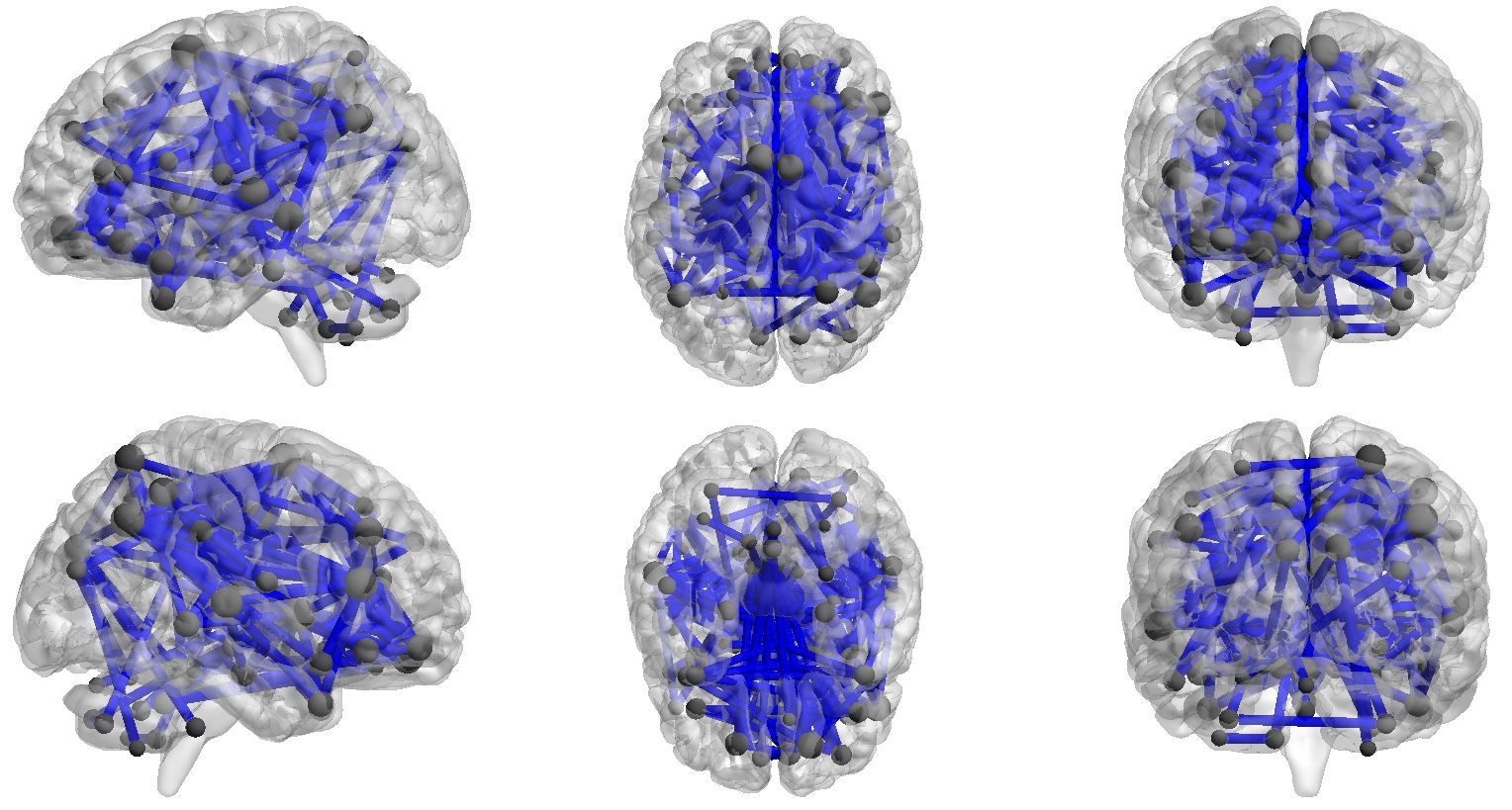


**Supplementary Figure 1:** Network of connections that are negatively correlated with CIAS-R scores. Gray spheres represent the centroid of each node and are scaled accordingly to the number of significant edges they are associated with. Only nodes with connections are shown.

**Supplementary Table 2.** List of connections within the identified network.

| CIAS-R positive |  |  |  |  |  |
| --- | --- | --- | --- | --- | --- |
| Node1 | Node2 | Region1 | Region2 | rho | p-value |
| Frontal_Mid_R | Frontal_Sup_L | F | F | -0.38502 | 0.003766 |
| Frontal_Inf_Orb_L | Frontal_Mid_R | F | F | -0.48918 | 0.000242 |
| Frontal_Inf_Orb_L | Frontal_Mid_Orb_R | F | F | -0.39219 | 0.0032 |
| Frontal_Inf_Orb_R | Frontal_Inf_Orb_L | F | F | -0.44465 | 0.00087 |
| Supp_Motor_Area_L | Frontal_Sup_R | F | F | -0.44043 | 0.000974 |
| Supp_Motor_Area_L | Frontal_Sup_Orb_R | F | F | -0.37045 | 0.005186 |
| Supp_Motor_Area_L | Frontal_Mid_R | F | F | -0.39988 | 0.002677 |
| Supp_Motor_Area_L | Frontal_Mid_Orb_R | F | F | -0.51562 | 0.000104 |
| Supp_Motor_Area_R | Frontal_Sup_R | F | F | -0.37045 | 0.005186 |
| Supp_Motor_Area_R | Frontal_Sup_Orb_L | F | F | -0.39537 | 0.002974 |
| Olfactory_R | Frontal_Mid_R | F | F | -0.38288 | 0.00395 |
| Frontal_Med_Orb_L | Frontal_Sup_Orb_R | F | F | -0.37334 | 0.004872 |
| Frontal_Med_Orb_R | Frontal_Sup_Orb_L | F | F | -0.37062 | 0.005167 |
| Frontal_Med_Orb_R | Supp_Motor_Area_L | F | F | -0.39086 | 0.003299 |
| Frontal_Med_Orb_R | Supp_Motor_Area_R | F | F | -0.41463 | 0.001879 |
| Frontal_Med_Orb_R | Frontal_Med_Orb_L | F | F | -0.37056 | 0.005173 |
| Insula_L | Frontal_Inf_Tri_R | I | F | -0.41805 | 0.001728 |
| Insula_L | Frontal_Med_Orb_R | I | F | -0.45072 | 0.000738 |
| Insula_R | Frontal_Sup_Orb_R | I | F | -0.37779 | 0.004421 |
| Insula_R | Frontal_Inf_Tri_R | I | F | -0.45622 | 0.000634 |
| Insula_R | Frontal_Med_Orb_R | I | F | -0.43644 | 0.001082 |
| Cingulum_Ant_L | Frontal_Sup_Orb_L | I | F | -0.46715 | 0.000466 |
| Cingulum_Ant_L | Frontal_Sup_Orb_R | I | F | -0.53511 | 5.33E-05 |
| Cingulum_Ant_L | Frontal_Mid_R | I | F | -0.38797 | 0.003524 |
| Cingulum_Ant_L | Frontal_Mid_Orb_R | I | F | -0.48109 | 0.00031 |
| Cingulum_Ant_L | Frontal_Inf_Tri_R | I | F | -0.424 | 0.001489 |
| Cingulum_Ant_L | Frontal_Inf_Orb_L | I | F | -0.37114 | 0.005109 |
| Cingulum_Ant_R | Frontal_Sup_Orb_L | I | F | -0.44482 | 0.000866 |
| Cingulum_Ant_R | Frontal_Sup_Orb_R | I | F | -0.37652 | 0.004546 |
| Cingulum_Ant_R | Frontal_Inf_Tri_R | I | F | -0.39746 | 0.002834 |
| Cingulum_Ant_R | Frontal_Inf_Orb_L | I | F | -0.3952 | 0.002986 |
| Cingulum_Mid_L | Frontal_Sup_Orb_R | I | F | -0.39722 | 0.002849 |
| Cingulum_Mid_L | Frontal_Mid_Orb_R | I | F | -0.42013 | 0.001641 |
| Cingulum_Mid_R | Frontal_Sup_Orb_L | I | F | -0.40977 | 0.002115 |
| Cingulum_Mid_R | Frontal_Sup_Orb_R | I | F | -0.3834 | 0.003905 |
| Cingulum_Mid_R | Supp_Motor_Area_R | I | F | -0.3734 | 0.004866 |
| Cingulum_Post_L | Frontal_Sup_Orb_R | I | F | -0.39318 | 0.003129 |
| Cingulum_Post_L | Frontal_Mid_R | I | F | -0.39202 | 0.003213 |
| Cingulum_Post_L | Frontal_Inf_Orb_R | I | F | -0.40503 | 0.00237 |
| Cingulum_Post_L | Supp_Motor_Area_L | I | F | -0.42539 | 0.001437 |
| Cingulum_Post_L | Supp_Motor_Area_R | I | F | -0.5273 | 7E-05 |
| Cingulum_Post_L | Insula_L | I | I | -0.45205 | 0.000712 |
| Cingulum_Post_L | Insula_R | I | I | -0.483 | 0.000292 |
| Cingulum_Post_L | Cingulum_Ant_L | I | I | -0.42672 | 0.00139 |
| Cingulum_Post_L | Cingulum_Ant_R | I | I | -0.40035 | 0.002648 |
| Cingulum_Post_L | Cingulum_Mid_L | I | I | -0.37762 | 0.004438 |
| Cingulum_Post_L | Cingulum_Mid_R | I | I | -0.39798 | 0.0028 |
| Cingulum_Post_R | Frontal_Inf_Orb_R | I | F | -0.39115 | 0.003278 |
| Cingulum_Post_R | Supp_Motor_Area_L | I | F | -0.38167 | 0.004059 |
| Cingulum_Post_R | Supp_Motor_Area_R | I | F | -0.44656 | 0.000826 |
| Cingulum_Post_R | Insula_L | I | I | -0.3941 | 3.06E-03 |
| Cingulum_Post_R | Insula_R | I | I | -0.39028 | 0.003343 |
| Cingulum_Post_R | Cingulum_Ant_L | I | I | -0.38195 | 0.004033 |
| ParaHippocampal_L | Supp_Motor_Area_R | T | F | -0.37079 | 0.005148 |
| ParaHippocampal_L | Insula_L | T | I | -0.37021 | 0.005212 |
| ParaHippocampal_L | Insula_R | T | I | -0.3908 | 0.003304 |
| ParaHippocampal_L | Hippocampus_R | T | T | -0.3801 | 0.004202 |
| ParaHippocampal_R | Insula_L | T | I | -0.49722 | 1.89E-04 |
| ParaHippocampal_R | Insula_R | T | I | -0.47999 | 0.00032 |
| Cuneus_L | Frontal_Sup_Orb_R | O | F | -0.3749 | 0.00471 |
| Cuneus_R | Frontal_Inf_Tri_R | O | F | -0.37802 | 0.004399 |
| Occipital_Mid_R | Supp_Motor_Area_L | O | F | -0.43389 | 0.001156 |
| Parietal_Sup_R | Frontal_Sup_Medial_L | P | F | -0.41105 | 0.002051 |
| Parietal_Sup_R | Frontal_Sup_Medial_R | P | F | -0.47791 | 0.00034 |
| Parietal_Sup_R | Cingulum_Post_L | P | I | -0.38433 | 0.003825 |
| Parietal_Sup_R | Cuneus_L | P | O | -0.3753 | 0.004669 |
| Parietal_Sup_R | Parietal_Sup_L | P | P | -0.40908 | 0.002151 |
| Parietal_Inf_L | Supp_Motor_Area_L | P | F | -0.37426 | 0.004776 |
| Parietal_Inf_R | Frontal_Inf_Oper_L | P | F | -0.39034 | 0.003339 |
| Parietal_Inf_R | Rolandic_Oper_L | P | F | -0.41752 | 0.00175 |
| Parietal_Inf_R | Rolandic_Oper_R | P | F | -0.46021 | 0.000567 |
| Parietal_Inf_R | Supp_Motor_Area_L | P | F | -0.47484 | 0.000373 |
| Parietal_Inf_R | Cingulum_Ant_L | P | I | -0.38866 | 0.003469 |
| Parietal_Inf_R | Cingulum_Mid_L | P | I | -0.3812 | 0.004101 |
| SupraMarginal_L | Cingulum_Post_L | P | I | -0.38224 | 0.004007 |
| SupraMarginal_R | Frontal_Inf_Oper_L | P | F | -0.37432 | 0.00477 |
| SupraMarginal_R | Cingulum_Post_L | P | I | -0.44558 | 0.000849 |
| Angular_L | Frontal_Mid_R | P | F | -0.37582 | 0.004616 |
| Angular_L | Rolandic_Oper_L | P | F | -0.39601 | 0.002931 |
| Angular_L | Supp_Motor_Area_L | P | F | -0.42406 | 0.001487 |
| Angular_L | Supp_Motor_Area_R | P | F | -0.49746 | 0.000187 |
| Angular_L | Cingulum_Mid_R | P | I | -0.4181 | 0.001725 |
| Angular_L | Parietal_Inf_R | P | P | -0.3967 | 0.002884 |
| Angular_L | SupraMarginal_L | P | P | -0.40052 | 0.002638 |
| Angular_R | Rolandic_Oper_L | P | F | -0.45645 | 0.00063 |
| Angular_R | Rolandic_Oper_R | P | F | -0.42458 | 0.001467 |
| Angular_R | Supp_Motor_Area_L | P | F | -0.55859 | 2.25E-05 |
| Angular_R | Supp_Motor_Area_R | P | F | -0.49826 | 0.000182 |
| Angular_R | Insula_L | P | I | -0.43198 | 0.001214 |
| Angular_R | Insula_R | P | I | -0.45575 | 0.000643 |
| Angular_R | Cingulum_Ant_L | P | I | -0.42759 | 0.001359 |
| Angular_R | Cingulum_Mid_L | P | I | -0.41411 | 0.001904 |
| Angular_R | Cingulum_Mid_R | P | I | -0.39786 | 0.002807 |
| Angular_R | Cuneus_R | P | O | -0.3956 | 0.002958 |
| Precuneus_L | Frontal_Inf_Orb_R | P | F | -0.37652 | 0.004546 |
| Precuneus_L | Supp_Motor_Area_R | P | F | -0.41249 | 0.00198 |
| Precuneus_L | Parietal_Sup_R | P | P | -0.42071 | 0.001617 |
| Precuneus_R | Frontal_Inf_Tri_L | P | F | -0.40648 | 0.00229 |
| Precuneus_R | Frontal_Inf_Tri_R | P | F | -0.48739 | 0.000256 |
| Precuneus_R | Frontal_Inf_Orb_R | P | F | -0.45431 | 0.000669 |
| Precuneus_R | Rolandic_Oper_R | P | F | -0.40283 | 0.002498 |
| Precuneus_R | Supp_Motor_Area_L | P | F | -0.43973 | 0.000992 |
| Precuneus_R | Supp_Motor_Area_R | P | F | -0.45049 | 0.000743 |
| Precuneus_R | Insula_R | P | I | -0.40468 | 0.00239 |
| Precuneus_R | Parietal_Sup_R | P | P | -0.41035 | 0.002086 |
| Precuneus_R | SupraMarginal_L | P | P | -0.40023 | 0.002656 |
| Precuneus_R | SupraMarginal_R | P | P | -0.3701 | 0.005225 |
| Putamen_R | Frontal_Med_Orb_R | S | F | -0.40312 | 0.00248 |
| Putamen_R | Amygdala_L | S | T | -0.41359 | 0.001928 |
| Pallidum_L | Cingulum_Post_L | S | I | -0.37247 | 0.004965 |
| Pallidum_L | Hippocampus_R | S | T | -0.38982 | 0.003379 |
| Pallidum_R | Cingulum_Ant_L | S | I | -0.40503 | 0.00237 |
| Pallidum_R | Cingulum_Post_L | S | I | -0.4417 | 0.000941 |
| Pallidum_R | Cingulum_Post_R | S | I | -0.40781 | 0.002218 |
| Pallidum_R | ParaHippocampal_L | S | T | -0.37964 | 0.004245 |
| Pallidum_R | ParaHippocampal_R | S | T | -0.37189 | 0.005027 |
| Thalamus_L | Cingulum_Post_R | S | I | -0.38462 | 0.0038 |
| Heschl_L | Angular_R | T | P | -0.40896 | 0.002157 |
| Heschl_R | Frontal_Inf_Tri_R | T | F | -0.42794 | 0.001347 |
| Heschl_R | Frontal_Med_Orb_R | T | F | -0.38155 | 0.004069 |
| Heschl_R | Parietal_Inf_R | T | P | -0.39398 | 0.003071 |
| Heschl_R | Angular_R | T | P | -0.40625 | 0.002303 |
| Temporal_Sup_L | Frontal_Inf_Tri_R | T | F | -0.38028 | 0.004186 |
| Temporal_Sup_L | Frontal_Med_Orb_R | T | F | -0.37409 | 0.004794 |
| Temporal_Sup_L | Cingulum_Post_L | T | I | -0.38901 | 0.003441 |
| Temporal_Sup_L | ParaHippocampal_R | T | T | -0.38236 | 0.003997 |
| Temporal_Sup_L | Angular_R | T | P | -0.47345 | 0.000388 |
| Temporal_Sup_L | Precuneus_R | T | P | -0.38357 | 0.00389 |
| Temporal_Sup_R | Frontal_Inf_Tri_R | T | F | -0.39855 | 0.002762 |
| Temporal_Sup_R | Cingulum_Post_L | T | I | -0.44893 | 0.000775 |
| Temporal_Sup_R | ParaHippocampal_R | T | T | -0.41157 | 0.002026 |
| Temporal_Sup_R | Angular_L | T | P | -0.39578 | 0.002946 |
| Temporal_Sup_R | Angular_R | T | P | -0.47432 | 0.000378 |
| Temporal_Sup_R | Precuneus_L | T | P | -0.37848 | 0.004354 |
| Temporal_Sup_R | Precuneus_R | T | P | -0.4336 | 0.001164 |
| Temporal_Pole_Sup_L | Frontal_Sup_Orb_R | T | F | -0.38352 | 0.003895 |
| Temporal_Pole_Sup_L | Frontal_Mid_R | T | F | -0.39532 | 0.002978 |
| Temporal_Pole_Sup_L | Frontal_Inf_Tri_R | T | F | -0.45766 | 0.000609 |
| Temporal_Pole_Sup_L | Insula_R | T | I | -0.37513 | 0.004686 |
| Temporal_Pole_Sup_R | Frontal_Sup_Orb_R | T | F | -0.47525 | 0.000368 |
| Temporal_Pole_Sup_R | Frontal_Inf_Tri_R | T | F | -0.41359 | 0.001928 |
| Temporal_Pole_Sup_R | Angular_R | T | P | -0.40943 | 0.002133 |
| Temporal_Pole_Sup_R | Temporal_Pole_Sup_L | T | T | -0.39185 | 0.003226 |
| Temporal_Mid_L | Frontal_Inf_Tri_L | T | F | -0.39329 | 0.003121 |
| Temporal_Mid_L | Supp_Motor_Area_L | T | F | -0.46721 | 0.000465 |
| Temporal_Mid_L | Supp_Motor_Area_R | T | F | -0.4321 | 0.001211 |
| Temporal_Mid_L | Insula_L | T | I | -0.38762 | 0.003551 |
| Temporal_Mid_L | Insula_R | T | I | -0.3974 | 0.002838 |
| Temporal_Mid_L | Parietal_Sup_R | T | P | -0.44234 | 0.000925 |
| Temporal_Mid_L | Precuneus_R | T | P | -0.40029 | 0.002652 |
| Temporal_Mid_L | Temporal_Sup_L | T | T | -0.39699 | 0.002864 |
| Temporal_Mid_L | Temporal_Sup_R | T | T | -0.4284 | 0.001332 |
| Temporal_Mid_R | Supp_Motor_Area_L | T | F | -0.37814 | 0.004388 |
| Temporal_Mid_R | Insula_L | T | I | -0.37924 | 0.004283 |
| Temporal_Mid_R | Insula_R | T | I | -0.38253 | 0.003981 |
| Temporal_Mid_R | Parietal_Sup_R | T | P | -0.40827 | 0.002194 |
| Temporal_Mid_R | Temporal_Sup_R | T | T | -0.38392 | 0.00386 |
| Temporal_Pole_Mid_L | Frontal_Inf_Tri_R | T | F | -0.39826 | 0.002781 |
| Temporal_Pole_Mid_L | Insula_L | T | I | -0.42157 | 0.001582 |
| Temporal_Pole_Mid_L | Insula_R | T | I | -0.41805 | 0.001728 |
| Temporal_Pole_Mid_L | Temporal_Sup_R | T | T | -0.39416 | 0.003059 |
| Temporal_Pole_Mid_R | Frontal_Inf_Tri_R | T | F | -0.41006 | 0.002101 |
| Temporal_Pole_Mid_R | Insula_L | T | I | -0.38103 | 0.004116 |
| Temporal_Pole_Mid_R | Insula_R | T | I | -0.43892 | 0.001013 |
| Temporal_Pole_Mid_R | Temporal_Sup_L | T | T | -0.43528 | 0.001115 |
| Temporal_Pole_Mid_R | Temporal_Sup_R | T | T | -0.41689 | 0.001778 |
| Temporal_Inf_L | Supp_Motor_Area_L | T | F | -0.42233 | 0.001553 |
| Temporal_Inf_L | Supp_Motor_Area_R | T | F | -0.39566 | 0.002954 |
| Temporal_Inf_L | Precuneus_R | T | P | -0.40121 | 0.002595 |
| Cerebelum_Crus2_L | Caudate_L | C | S | -0.47195 | 0.000405 |
| Cerebelum_Crus2_R | Cingulum_Post_R | C | I | -0.41868 | 0.001701 |
| Cerebelum_Crus2_R | Cerebelum_Crus2_L | C | C | -0.43684 | 0.00107 |
| Cerebelum_3_L | Frontal_Sup_Orb_L | C | F | -0.38456 | 0.003805 |
| Cerebelum_3_L | Frontal_Sup_Orb_R | C | F | -0.39277 | 0.003158 |
| Cerebelum_3_L | Pallidum_R | C | S | -0.39445 | 0.003039 |
| Cerebelum_3_R | Pallidum_L | C | S | -0.38693 | 0.003607 |
| Cerebelum_4_5_L | Cuneus_L | C | O | -0.3753 | 0.004669 |
| Cerebelum_4_5_L | Cuneus_R | C | O | -0.4365 | 0.00108 |
| Cerebelum_6_L | Occipital_Mid_R | C | O | -0.37449 | 0.004752 |
| Cerebelum_8_R | Cuneus_R | C | O | -0.40081 | 0.00262 |
| Cerebelum_9_L | Supp_Motor_Area_R | C | F | -0.42325 | 0.001517 |
| Cerebelum_9_L | Cerebelum_7b_L | C | C | -0.38294 | 0.003945 |
| Cerebelum_10_R | Cuneus_L | C | O | -0.38959 | 0.003396 |
| Cerebelum_10_R | Cuneus_R | C | O | -0.46431 | 0.000505 |
| Vermis_1_2 | Frontal_Sup_Orb_L | C | F | -0.46322 | 0.000521 |
| Vermis_1_2 | Frontal_Sup_Orb_R | C | F | -0.46379 | 0.000513 |
| Vermis_1_2 | Frontal_Mid_Orb_R | C | F | -0.39641 | 0.002903 |
| Vermis_1_2 | Frontal_Sup_Medial_R | C | F | -0.38375 | 0.003875 |
| Vermis_1_2 | Cingulum_Post_R | C | I | -0.37611 | 0.004587 |
| Vermis_1_2 | Pallidum_R | C | S | -0.42337 | 0.001513 |
| Vermis_7 | Caudate_L | C | S | -0.38045 | 0.00417 |
| Vermis_9 | Caudate_L | C | S | -0.40844 | 0.002184 |
| Vermis_9 | Vermis_1_2 | C | C | -0.42175 | 0.001576 |
| Vermis_10 | Frontal_Sup_Orb_L | C | F | -0.42614 | 0.00141 |
| Vermis_10 | Frontal_Sup_Medial_L | C | F | -0.45147 | 0.000723 |
| Vermis_10 | Frontal_Med_Orb_L | C | F | -0.39271 | 0.003162 |
| Vermis_10 | Rectus_R | C | F | -0.51324 | 0.000112 |
| *F: frontal lobe; T: temporal lobe; P: parietal lobe; O: occipital lobe; I: insula and cingulate cortex; S: subcortical; C: cerebellum | | | | | |
| **Sup: superior; Orb: orbital; Mid: middle; Inf: inferior; Oper: opercular/operculum; Tri: triangular; Supp: supplementary; Med: medial; Ant: anterior; Post: posterior; Cerebelum: cerebellum | | | | | |

*Global distribution of involved edges*

To get a better understanding of how these connections are distributed, we followed Fornito, Yoon, Zalesky, Bullmore, and Carter (2011) and Hong et al. (2013), and categorized each AAL region within each network as belonging to seven regional subgroupings: frontal, temporal, parietal, occipital, insula and cingulate gyri, subcortical, and cerebellum. The majority of edges in the identified network involves connections between (1) frontal regions and insula & cingulate gryi (~14%), most of which involves connections between the orbital frontal lobe and cingulate cortex; and (2) frontal and parietal regions (~12%), which includes connections between the supplementary motor area and angular gyrus as well as the precuneus. Connections involving the occipital, subcortical, and the cerebellum showed relatively smaller percentage of the network. The proportions of inter-regional connections of each network is illustrated in Supplementary Figure 2.


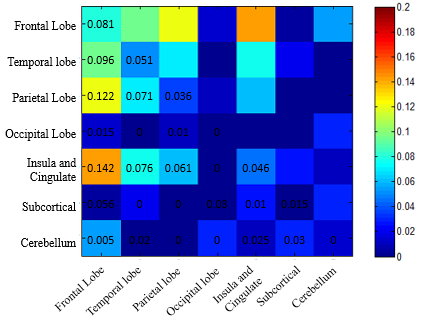


**Supplementary Figure 2:** Proportion of edges that are positively and negatively correlated with internet addiction tendency among pairs of regional subgroupings. The proportions were calculated by dividing the number of edges between (or within) pairs of regions with the total number of edges identified in each network.

*Maximally affected nodes*

Due to the large number of edges identified, we followed Finn et al. (2014), and identified nodes that have a high “sum of CIAS-R-correlated edges” in order to focus our analysis on regions where connections are maximally related to internet addiction tendency. The sum of CIAS-R-correlated edges of a node was defined as the total number of its edges. This method will enable us to identify nodes where connections are most likely to be altered by internet addiction tendency. The following Table lists the nodes that are maximally affected, and shows nodes that have at least a sum of CIAS-R-correlated edges of at least 7.

**Supplementary Table 3.** Node level analysis of internet addiction tendency

| AAL Node | MNI coordinate | | | Sum of correlated edges |
| --- | --- | --- | --- | --- |
| Cingulum_Post_L | -4.85 | -42.92 | 24.67 | 18 |
| Supp_Motor_Area_L | -5.32 | 4.85 | 61.38 | 16 |
| Angular_R | 45.51 | -59.98 | 38.63 | 15 |
| Supp_Motor_Area_R | 8.62 | 0.17 | 61.85 | 14 |
| Insula_R | 39.02 | 6.25 | 2.08 | 14 |
| Precuneus_R | 9.98 | -56.05 | 43.77 | 14 |
| Frontal_Sup_Orb_R | 18.49 | 48.1 | -14.02 | 13 |
| Frontal_Inf_Tri_R | 50.33 | 30.16 | 14.17 | 13 |
| Insula_L | -35.13 | 6.65 | 3.44 | 11 |
| Cingulum_Ant_L | -4.04 | 35.4 | 13.95 | 11 |
| Temporal_Sup_R | 58.15 | -21.78 | 6.8 | 11 |
| Cingulum_Post_R | 7.44 | -41.81 | 21.87 | 10 |
| Frontal_Med_Orb_R | 8.16 | 51.67 | -7.13 | 9 |
| Parietal_Sup_R | 26.11 | -59.18 | 62.06 | 9 |
| Temporal_Mid_L | -55.52 | -33.8 | -2.2 | 9 |
| Frontal_Sup_Orb_L | -16.56 | 47.32 | -13.31 | 8 |
| Frontal_Mid_R | 37.59 | 33.06 | 34.04 | 8 |
| Parietal_Inf_R | 46.46 | -46.29 | 49.54 | 8 |
| Angular_L | -44.14 | -60.82 | 35.59 | 8 |
| Temporal_Sup_L | -53.16 | -20.68 | 7.13 | 8 |
| Pallidum_R | 21.2 | 0.18 | 0.23 | 7 |
| Vermis_1_2 | 0.76 | -38.79 | -20.05 | 7 |

*Sup: superior; Orb: orbital; Mid: middle; Inf: inferior; Oper: opercular/operculum; Tri: triangular; Supp: supplementary; Med: medial; Ant: anterior; Post: posterior; Cerebelum: cerebellum
